# Genome-Wide Identification and Expression Analysis of the R2R3-MYB Transcription Factor Family Revealed Their Potential Roles in the Flowering Process in Longan (*Dimocarpus longan*)

**DOI:** 10.3389/fpls.2022.820439

**Published:** 2022-03-25

**Authors:** Qinchang Chen, Xiaodan Zhang, Yaxue Fang, Baiyu Wang, Shaosi Xu, Kai Zhao, Jisen Zhang, Jingping Fang

**Affiliations:** ^1^College of Life Sciences, Fujian Normal University, Fuzhou, China; ^2^Center of Engineering Technology Research for Microalgae Germplasm Improvement of Fujian, Southern Institute of Oceanography, Fujian Normal University, Fuzhou, China; ^3^Department of Plant Biology, University of Illinois at Urbana-Champaign, Urbana, IL, United States; ^4^Center for Genomics and Biotechnology, Fujian Provincial Key Laboratory of Haixia Applied Plant Systems Biology, Key Laboratory of Genetics, Breeding and Multiple Utilization of Crops, Ministry of Education, Fujian Agriculture and Forestry University, Fuzhou, China

**Keywords:** *Dimocarpus longan*, *R2R3-MYB*, flower development, KClO_3_, qRT-PCR

## Abstract

Longan (*Dimocarpus longan* Lour.) is a productive fruit crop with high nutritional and medical value in tropical and subtropical regions. The *MYB* gene family is one of the most widespread plant transcription factor (TF) families participating in the flowering regulation. However, little is known about the MYB TFs involved in the flowering process in longan and its regulatory network. In this study, a total of 119 *DlR2R3-MYB* genes were identified in the longan genome and were phylogenetically grouped into 28 subgroups. The groupings were supported by highly conserved gene structures and motif composition of *DlR2R3-MYB* genes in each subgroup. Collinearity analysis demonstrated that segmental replications played a more crucial role in the expansion of the *DlR2R3-MYB* gene family compared to tandem duplications, and all tandem/segmental duplication gene pairs have evolved under purifying selection. Interspecies synteny analysis among longan and five representative species implied the occurrence of gene duplication events was one of the reasons contributing to functional differentiation among species. RNA-seq data from various tissues showed *DlR2R3-MYB* genes displayed tissue-preferential expression patterns. The pathway of flower development was enriched with six *DlR2R3-MYB* genes. *Cis*-acting element prediction revealed the putative functions of *DlR2R3-MYB* genes were related to the plant development, phytohormones, and environmental stresses. Notably, the orthologous counterparts between Arabidopsis and longan R2R3-MYB members tended to play conserved roles in the flowering regulation and stress responses. Transcriptome profiling on off-season flower induction (FI) by KClO_3_ indicated two up-regulated and four down-regulated *DlR2R3-MYB* genes involved in the response to KClO_3_ treatment compared with control groups. Additionally, qRT-PCR confirmed certain genes exhibited high expression in flowers/flower buds. Subcellular localization experiments revealed that three predicted flowering-associated MYB proteins were localized in the nucleus. Future functional studies on these potential candidate genes involved in the flowering development could further the understanding of the flowering regulation mechanism.

## Introduction

Myeloblastosis (MYB) proteins were first identified in the avian myeloblastosis virus (AMV) (Klempnauer et al., 1982), but have since been found in all eukaryote genomes and belong to one of the largest transcription factor (TF) gene families in plants. The first plant *MYB* gene identified from *Zea mays*, known as *COLORED1* (*C1*), was involved in the regulation of anthocyanin biosynthesis (Paz-Ares et al., 1987). The MYB TF contains a highly conserved MYB DNA-binding domain (DBD) in the N-terminal region that is usually composed of 1–4 serial imperfect repeat sequences. Each repeat sequence covers about 50–55 amino acid residues including three regularly spaced tryptophan (W) residues [-W-(X_19_)-W-(X_19_)-W…-F/I-(X-_18_)-W–(X-_18_)-W-], which fold into three α-helices (Kanei-Ishii et al., 1990; Kranz et al., 2000). The second and third helices form a helix-turn-helix (HTH) structure, and the third α-helix directly participates in binding with DNA (Ogata et al., 1992; Williams and Grotewold, 1997; Jia et al., 2004). MYB TFs can be divided into four categories based on the number of MYB repeats in their DBDs: 4R-MYB (four repeats), R1R2R3-MYB (three repeats), R2R3-MYB (two repeats), and 1RMYB (one repeat). The *R2R3-MYB* genes have received significant attention because they are massively expanded in the plant lineage and involved in regulating a wide array of plant-specific biological processes, such as primary and secondary metabolism, response to plant hormones and environmental stresses, disease resistance and leaf morphogenesis (Roy, 2016; Millard et al., 2019a; Jiang and Rao, 2020). The evolutionary history of the *R2R3-MYB* (*2R-MYBs*) gene family throughout the eukaryotic kingdom remains unknown (Du et al., 2015). The “loss” model proposes that *R2R3-MYB* genes potentially evolved from *R1R2R3-MYB* progenitor genes through the loss of the R1 repeat sequence (Rosinski and Atchley, 1998; Braun and Grotewold, 1999). In contrast, the more convincing “gain” model hypothesizes that *R1R2R3-MYB* genes evolved from the *R2R3-MYB*s via intragenic structural domain duplication (Jiang et al., 2004; Du et al., 2015).

With the rapid development of high-throughput sequencing technologies, genome-wide identification of R2R3-MYB proteins has been achieved in many plant species, for instance, *Arabidopsis thaliana* (Stracke et al., 2001; Dubos et al., 2010), *Medicago truncatula* (Li et al., 2019), *Casuarina equisetifolia* (Wang et al., 2021), *Solanum tuberosum* (Sun et al., 2019), *Oryza sativa* (Katiyar et al., 2012), and *Capsicum annuum* (Wang et al., 2020). In general, the number of R2R3-MYBs in plants ranged in size from 70 to 285, and these *MYB* genes evolved mainly based on the natural evolution of organisms and the recombination and amplification of genomes (Chang and Duda, 2012). The highest number of R2R3-MYBs (285) was found in the banana genome (Pucker et al., 2020). Respectively, 244, 192, 157, and 134 *R2R3-MYB* genes have been identified in the soybean (Du et al., 2012b), populus (Wilkins et al., 2009), maize (Du et al., 2012a), and grape (Wong et al., 2016) based on previous studies. In addition, the lowest number of *R2R3-MYB* genes, a total of 70, were identified in the sugar beet genome (Stracke et al., 2014).

R2R3-MYB TFs as regulatory proteins play crucial roles in plant growth and development (Kranz et al., 2000; Dubos et al., 2010). With regards to the flower development, *AtMYB33* and *AtMYB65* were microRNA-regulated genes that could promote anther and pollen development (Millar and Gubler, 2005). *AtMYB80* regulated pollen development and programed cell death in the chorioallantoic layer (Phan et al., 2011). *AtMYB106* regulated the flowering time of plants by inhibiting the expression of the *Flowering Locus T* (*AtFT*) gene, which is a negative regulator in plant flowering development (Hong et al., 2021). In chrysanthemum, *CmMYB2* interacted with CmBBX24 to influence the synthesis of gibberellin and ultimately regulated flowering (Zhu et al., 2020). In addition, the R2R3-MYB TFs are known to be widely involved in the regulation of plant specialized metabolism. Previous studies showed that several *AtR2R3-MYB* genes such as *AtMYB11*/*PFG1*, *AtMYB12*/*PFG1*, *AtMYB111*/*PFG3*, *AtMYB21*, and *AtMYB24* were involved in regulating the flavonol biosynthesis pathway in Arabidopsis (Stracke et al., 2007; Dubos et al., 2010; Shan et al., 2020). Overexpression of a flavonol regulator, *MtMYB134*, in hairy roots of *Medicago truncatula* could enhance the biosynthesis of various flavonol derivatives (Naik et al., 2021). *AtMYB75*/*PAP1*, *AtMYB90*/*PAP2*, *AtMYB113*, and *AtMYB114* were identified as regulators of anthocyanin biosynthesis (Teng et al., 2005; Gonzalez et al., 2008). R2R3-MYB family genes are also widely involved in the plant responses to biotic and abiotic stress besides participating in various physiological activities. *AtMYB15* and *AtMYB44* could enhance drought and salt tolerance by regulating stomatal closure under abscisic acid (ABA) treatments (Jung et al., 2008; Ding et al., 2009). *AtMYB96* increased resistance to low temperature through promoting the expression of C-repeat binding factor (*CBF*) (Lee and Seo, 2015). The overexpression of *AtMYB96* also enhanced drought tolerance by mediating ABA signaling and regulating the target gene Lipid transfer protein 3 (*LTP3*) (Seo et al., 2009). In other species, *LcMYB2* promoted seed germination and root growth under drought stress (Zhao et al., 2019). Overexpression of *ZmMYB3R* improved tolerance to drought and salt stress in transgenic Arabidopsis plants (Wu et al., 2019).

Longan (*Dimocarpus longan* Lour., 2n = 2x = 30) is an important tropical and subtropical fruit tree of the *Sapindaceae* family in the Sapindales order, widely grown in southern China and Southeast Asia (Matsumoto, 2006). As one of the cradles of longan, China currently ranks first in the longan cultivation acreage and yields in the world accounting for 50% of global longan production. Longan has high nutritional and medicinal values and mainly uses its palatable and juicy fruit for commercial purposes (Rangkadilok et al., 2005; Zhang et al., 2020). Flower induction (FI) is an important regulatory process in the transition from vegetative to reproductive growth in plants. Environmental factors like low temperature, drought, and oxidative stress can regulate the flowering of many plants. Potassium chlorate (KClO_3_) treatment is one of the most effective ways that widely apply in longan production to induce off-season flowering (Huang et al., 2021). To the best of our knowledge, KClO_3_-induced flowering has only been utilized in longan (Huang et al., 2021). Studies on the physiological mechanism of KClO_3_-mediated FI showed that KClO_3_ could increase the content of hormones such as cytokinin (CK) and salicylic acid (SA) in longan, which are closely related to plant flower formation (Sringarm et al., 2009). The KClO_3_ treatment up-regulated CK concentration together with *DlFT1* expression in mature leaves (Winterhagen et al., 2020). In addition, KClO_3_ treatment caused changes in the carbon to nitrogen ratio by decreasing the starch content and increasing the soluble sugar, sucrose and soluble amino acid content, which provided nutrients for the flower bud differentiation (Chang, 2010). A regulatory model of FI by KClO_3_ in longan has been proposed: KClO_3_ may be reduced to chlorite and hypochlorite under the action of nitrate reductase (NR) and nitrite reductase (NiR) to directly induce stress responses, leading to an increase in CK content and expression of flowering-related genes (Borges et al., 2004; Cho et al., 2017). Thus far, several flowering-related genes have been identified and analyzed in longan such as *flowering locus T* (*DlFT*), *gigantea* (*DlGI*), *early flowering 4* (*DlELF4*), *flavin-binding, kelch repeat, F-box 1* (*DlFKF1*), *short vegetative phase* (*DlSVP*) and *APETALA1-like (DlAP1*) genes (Winterhagen et al., 2013; Jia et al., 2014; Huang F. N. et al., 2017; Waheed et al., 2020) but the molecular mechanism underlying longan FI and floral organ development is largely unknown.

The *R2R3-MYB* genes comprise one of the largest TF families in longan. Previous studies have identified 35 (Zheng et al., 2020) and 98 (Lin et al., 2017) *R2R3-MYB* genes based on transcriptome data and the Next Generation Sequencing (NGS)-based assembled genome sequences, respectively. Recently, a high-quality chromosome-level genome of longan “Shixia” (“SX”) was assembled by our team (NCBI GenBank: JAIFKA000000000.1), which will help uncover the distribution, evolution and function of genome-wide R2R3-MYB TFs. By combining PacBio long reads, HiC data and genetic maps, the longan genome was assembled into 483.32 Mb with a contig N50 size of 763.91 kb, which were further anchored onto 15 pseudochromosomes with a 99.30% anchored rate. Benchmarking Universal Single-Copy Orthologs (BUSCO) assessment of the genome was 94.6%, which was an indicator of good quality assembly. Transcriptome dynamics revealed that six associated genes including one *MYB* transcript factor (*Dil.04g005810.2.t1*) were closely related to the flowering time under the KClO_3_ treatment (unpublished data). Studying the *MYB* gene expression profiles associated with off-season FI by KClO_3_ in longan will provide a deeper understanding of the molecular regulation mechanism of the flowering process in woody plants.

In this present study, we identified 119 *DlMYB* genes from the “Shixia” longan genome sequences and divided them into 28 subgroups. The *DlMYB* gene sequences were further investigated for their characterization, phylogenetic relationship, gene structure, subcellular localization, conserved motifs, *cis*-acting elements, chromosome distribution, synteny analysis, selection pressure, and gene duplication. In addition, the RNA-seq data showed expression patterns of *DlMYBs* in nine different tissues and in flower bud tissues treated with KClO_3_ at different stages which assisted us in determining the potential role of *DlMYBs* in longan flowering. The qRT-PCR method was used to verify the expression levels of the selected *MYB* genes in longan tissues. Moreover, three potential flowering-associated *MYB* genes were further experimentally validated by their subcellular localization in Arabidopsis protoplasts. This is the first systematic report on the genome-wide characterization of the *MYB* gene family in longan. This study will help investigate members of the R2R3-MYB family involved in the flowering process in longan and the regulatory network, which will pave the way for future functional studies and candidate gene selection for genetic improvement in longan.

## Materials and Methods

### Plant Materials and KClO_3_ Treatments

Longan cultivar trees (“Shixia,” abbreviated “SX”) about 4− to 6− year-olds in Maoming, Guangdong, China, were selected for treatment with white KClO_3_ powder of >99% purity in 2016. The apical buds were collected under controlled conditions and KClO_3_ treatment from longan trees at ten time points with three biological replications including 0 day after treatment (DAT) (November 18th, 2016), 5 DAT (November 23th, 2016), 10 DAT (November 28th, 2016), 15 DAT (December 3rd, 2016), 20 DAT (December 8th, 2016), 25 DAT (December 13th, 2016), 30 DAT (December 18th, 2016), 35 DAT (December 23th, 2016), 41 DAT (December 29th, 2016) and 54 DAT (January 11th, 2017). Immediately after harvesting, all tissue samples were frozen in liquid nitrogen and then stored at –80°C prior to the RNA isolation.

### Illumina Library Construction and RNA Sequencing

Total RNAs were extracted from collected samples with three biological replicates using the RNAprep Pure Plant Kit (DP432, TIANGEN Biotech, Beijing, China) according to the manufacturer’s protocol. The quality of total RNAs was verified on an Agilent 2100 Bioanalyzer (Plant RNA Nano Chip, Agilent, CA, United States). After quantity and quality determination, RNA-Seq libraries were constructed using the Illumina^®^ TruSeq*™* RNA Sample Preparation Kit [RS-122-2001(2), Illumina] and then subjected to Illumina transcriptome short reads sequencing on Illumina X Ten platform with paired-end (PE) 150 nt mode (San Diego, CA, United States).

### Public Data Retrieval

The genomic data of *Dimocarpus longan* “Shixia” (GenBank accession No.: JAIFKA000000000.1), *Litchi chinensis* (GenBank accession No.: JAIUGE000000000.1), *Oryza sativa* (GenBank accession No.: RPSM01000000), and *Zea mays* (GenBank accession No.: CABHLF010000000) were downloaded from the National Center for Biotechnology Information (NCBI). The data for *Xanthoceras sorbifolium* was downloaded from GigaDB (Sneddon et al., 2012). The data for *Arabidopsis thaliana* R2R3-MYB genes were retrieved from The Arabidopsis Information Resource (TAIR) database. The RNA-seq data of “Sijimi” (“SJM”) longan were downloaded from the NCBI (GenBank accession No.: PRJNA329283), including nine tissues (roots, stems, leaves, flower buds, flowers, young fruits, pulps, pericarps, and seeds) from a previous study (Lin et al., 2017).

### Identification of Longan R2R3-MYB Family Genes

The Hidden Markov Model (HMM) profiles for the Myb_DNA-binding domain (PF00249) and SWIRM domain (PF04433) were downloaded from the Pfam protein family database. The specific identification method of *R2R3-MYB* genes was as follows. The largest number of R2R3-MYB proteins were identified based on the HMM profiles of the MYB domain and SWIRM domain by using HMMER 3.0 with *E*-values ≤ 0.01 threshold (Potter et al., 2018). Gene sequences containing MYB domains but no SWIRM domain were considered as putative *MYB* genes. All longan *R2R3-MYB* genes were verified using *AtR2R3-MYB* gene sequences as target sequences by BLASTP, with the following parameters: *E*-value cut-off of 1e^–5^ and 30% identity. These sequences were manually inspected by the NCBI Conserved Domain Database (NCBI-CDD) and the SMART website to ensure that the genes contained two MYB domains. The full-length amino acid sequences of DlMYB proteins were submitted to the Expasy website (Gasteiger et al., 2003) to predict their molecular weights (MW), isoelectric points (PIs), hydrophilia, and instability. Detailed information on the *DlMYB* genes is listed in Supplementary Table 1. Multiple sequence alignments of the MYB domains were performed by Clustal X (2.1) (Larkin et al., 2007) with default parameters. The amino acid sequences in MYB domains were visualized by GeneDoc (Nicholas et al., 1997). Weblogo (Crooks et al., 2004) was used to display the sequence logos of R2 and R3 MYB domain repeats based on the multiple alignment files.

### Phylogenetic Analysis

The multiple sequence alignments of the full-length R2R3-MYB protein sequences from longan and Arabidopsis were performed by Muscle v3.8.1 (Edgar, 2004) with default parameters. The phylogenetic tree of R2R3-MYB proteins from both genomes was constructed using the Maximum Likelihood (ML) method of RAxML (Stamatakis, 2014), with the following parameters: JTT model and 1,000 bootstrap replications. The Evolview (Subramanian et al., 2019) was used to visualize the ML phylogenetic tree. Longan R2R3-MYB proteins were grouped according to the previously reported *AtR2R3-MYB* family classification (S1–S25) (Stracke et al., 2001; Dubos et al., 2010). Another ML phylogenetic tree of R2R3-MYB protein sequences from the longan genome was constructed using the same method.

### Motif Prediction, Gene Structure, and *Cis*-Acting Element Analysis

The MEME v5.3.3 (Multiple expectation maximization for Motif Elicitation) (Bailey et al., 2009) online tool was used to determine the conserved motifs in DlMYB amino acid sequences, with the following parameters: zero or one occurrence per sequence; maximum number of motif to find, 20; minimum width of motif, 6; and maximum width of motif, 200. Weblogo (Crooks et al., 2004) was used to display these conserved motifs.

The exon/intron structure patterns of *DlMYB* genes, including intron distribution patterns and intron-exon boundaries, were graphically visualized by the TBtools v1.087 (Chen et al., 2020), using the nucleotide sequences of *DlMYB* genes and CDSs (coding sequences). The 2-kb sequences upstream of each longan *R2R3-MYB* gene sequence were submitted to the PlantCARE database (Lescot et al., 2002) to predict *cis*-acting elements. The predicted results of gene structures and *cis*-acting elements were visualized by TBtools program (Chen et al., 2020).

### Chromosomal Locations and Synteny Analysis

The detailed information of the physical locations of *DlMYB* genes were generated based on the longan genome sequences (NCBI GenBank: JAIFKA000000000.1). The TBtools was used to exhibit chromosomal locations of *DlMYB* genes using the gene location visualization program. The Multiple Collinearity Scan toolkit (MCScanX) (Wang et al., 2012) and TBtools were used to analyze and visualize *DlMYB* gene replication events with *E*-value cut-off of < 1e^–10^. The Dual Systeny Plot program in TBtools (Chen et al., 2020) was adopted to elucidate the synteny relationship of orthologous *R2R3-MYB* genes between longan and other five species, including three dicots Arabidopsis, litchi and yellow horn, and two monocots rice and maize. The Ka/Ks (non-synonymous/synonymous substitution ratio) values were used to determine the selection pressure after replication. Ka/Ks > 1 signifies purified selection, Ka/Ks = 1 indicates neutral selection, and Ka/Ks < 1 represents positive selection (Hurst, 2002). The MS model in KaKs_calculator 2.0 (Wang et al., 2010) was used to calculate the values of Ka, Ks, and Ka/Ks of *MYB* duplicated gene pairs.

### Subcellular Localization Analysis

Subcellular localization was used to analyze the specific location of the *DlMYB* genes within the cell. The subcellular localization and nuclear localization signal (NLS) were predicted using the YLoc (Briesemeister et al., 2010) and NLStradamus online tool (Nguyen Ba et al., 2009), respectively. Three flower-preferential expressed *DlMYB* genes, *DlMYB16*, *DlMYB72*, and *DlMYB116* were selected for further experimental validation of their subcellular localizations. Total RNAs were extracted from leaves, flowers and flower buds of “Shixia” longan plants using the RNAprep pure Plant Kit (Tiangen, #DP432) according to the manufacturer’s protocol. The full-length coding sequences excluding stop codon of these three *R2R3-MYB* genes were amplified and cloned into the *Bsa*I/*Eco*31I restriction sites of the pBWA(V)HS-GLosgfp vector (Supplementary Figure 5; Sun et al., 2018). Briefly, the pBWA(V)HS-DlMYB-GFP (35S:GFP) recombinant vector and the nucleus marker vector pBWA(V)HS-Nucleus-mKATE (Shcherbo et al., 2007) were co-transformed in Arabidopsis protoplasts with PEG solution (40% W/V PEG 4000, 0.6 mol/L Mannitol, 0.1 mol/L calcium chloride, pH 5.8). The pBWA(V)HS-GFP empty vector was used as a control. After incubation in darkness at 28°C for 18–24 h, the subcellular distribution and localization of DlMYB-GFP were captured in protoplasts using the Nikon C2-ER laser scanning confocal microscope (LSCM) at a 488 nm excitation wavelength and a 510 nm emission wavelength. Meanwhile, colocalization of DlMYB-GFP and Nucleus-mKATE (NLS-mKate) was used to confirm the nuclear localization of MYB proteins with the mKATE protein at 561 nm excitation wavelength and 580 nm emission wavelength (Shcherbo et al., 2007; Zhao et al., 2017). The chlorophyll fluorescence was captured to confirm the localization of chloroplasts at 640 nm excitation wavelength and 675 nm emission wavelength. The primers used for PCR amplification are listed in Supplementary Table 2.

### Gene Expression Pattern Analysis by RNA-seq

RNA-seq data from nine tissues of “Sijimi” (“SJM”) longan (See more details in section “Public Data Retrieval”) were used to explore the expression patterns of *R2R3-MYB* genes at different developmental stages of the longan plant (Lin et al., 2017). The Fastp v0.20.1 software (Chen et al., 2018) was used to filter low-quality reads and adaptor sequences. High-quality clean reads were mapped to the “SX” longan reference genome sequences using HISAT2 v2.2.1 (Kim et al., 2015). The trimmed mean of *M* values (TMM) method was used to normalize the expression levels of *R2R3-MYB* genes in longan using edgR software (Robinson et al., 2010). The expression profiles of the *R2R3-MYB* genes in longan tissues were visualized by TBtools and the ratios were log_2_-transformed. Gene ontology (GO) annotations were conducted in the eggNOG-mapper (Huerta-Cepas et al., 2019) for the *DlMYB* genes. All 37,142 genes of longan were used as the reference set, and 119 *DlMYB* genes were taken as the test set. The annotation results of *DlMYB* genes were enriched and analyzed using the R package clusterProfiler (Yu et al., 2012), including three categories, cellular component (CC), biological process (BP), and molecular function (MF).

The RNA-seq data of “SX” under KClO_3_ treatment was also processed using the same method. The differentially expressed genes (DEGs) under KClO_3_ treatments and controlled conditions in longan were identified using the R package DESeq2 v3.11 (Love et al., 2014), with the threshold of |log2(fold-change)| ≥ 1 and a false discovery rate (FDR) ≤ 0.05. The DESeq2’s Likelihood Ratio Test was used to conduct the time course analysis to find genes which were significantly upregulated/downregulated over time in different treatments. The weighted gene co-expression network analysis (WGCNA) was conducted using the WGCNA v4.0.2 package (Langfelder and Horvath, 2008) based on DEGs with TMM ≥ 1 at least in one sample. Cytoscape v3.7.1 software (Shannon et al., 2003) was used to visualize the gene interaction networks of longan *R2R3-MYB* genes in modules. FLOweRing Interactive Database (FLOR-ID) of Arabidopsis (Bouché et al., 2016) was used to search flowering-related genes that may interact with *DlR2R3-MYB* genes. GO and KEGG enrichment analysis were performed using the R package clusterProfiler.

### Expression Validation by Quantitative Real-Time PCR

Eight *DlMYB* genes potentially involved in the flower development were selected for the quantitative real-time polymerase chain reaction (qRT-PCR) in five different tissues (leaves, pericarps, pulps, flowers, and flower buds) of “Shixia.” The total RNA in the samples were isolated using the TRNzol Universal (DP424, TIANGEN Biotech, Beijing, China) reagent according to the manufacturer’s instructions. The concentration and quality of the extracted RNAs was determined using Nanodrop 2000 spectrophotometer (Thermo Fisher Scientific, MA, United States) and gel electrophoresis. For cDNA synthesis, 0.5 μg RNA was reverse-transcribed using the M-MLV 4 First-Strand cDNA Synthesis Kit (MT403-01, Biomed, Beijing, China) following the supplier’s instructions. The cDNA was stored at –20°C for further use. Gene-specific primers were designed using the online tool primer3plus (Untergasser et al., 2007; Supplementary Table 2). The longan actin gene (*Dil.06g016430.1*) was used as the internal reference gene. The qRT-PCR amplification was conducted on a CFX Connect Real-Time PCR detection system (Bio-Rad, Hercules, CA, United States) with 2 × SYBR Green qPCR MasterMix (MT521, Biomed, Beijing, China). Three independent biological replicates and three technical repeats were taken. The reaction was performed using the protocol for this kit with the following minor modifications: 95°C for 30 s, 40 cycles of 95°C for 5 s, and 60°C for 30 s. The relative expression levels of *DlMYB* genes were further calculated using the 2^–ΔΔCT^ method (Livak and Schmittgen, 2001).

## Results

### Identification and Characterization of Longan *MYB* Genes

A total of 219 non-redundant *DlMYB* genes were identified in the longan reference genome, including 119 *R2R3-MYB* genes (2R-MYBs), 95 *MYB-like* repeats genes (1R-MYBs), three *R1R2R3-MYB* genes (3R-MYBs), and two *4RMYB* genes (4R-MYBs). The *R2R3-MYB* genes constituted the largest group of the MYB gene family in longan. The *R2R3-MYB* genes were renamed *DlMYB1* to *DlMYB119* based on their chromosomal locations in longan genome (Supplementary Table 1). The detailed information of *DlMYB* gene features, including coding sequences, protein sequences, chromosomal locations, protein sizes, molecular weights (MWs), isoelectric points (PIs), subcellular localizations, instability, and hydrophilia, were listed in Supplementary Tables 1, 3. The length of DlMYB proteins ranged from 134 (DlMYB38) to 661 (DlMYB55) amino acids (aa) with an average of 306 aa, and the MWs had a range of sizes from 15.7 (DlMYB55) to 75.8 (DlMYB38) KDa (mean 34.6). The PIs of the DlMYB proteins ranged from 4.91 (DlMYB32) to 10.60 (DlMYB23). All DlMYB protein hydrophilia values were negative, indicating that longan MYBs are hydrophilic.

**FIGURE 1:**
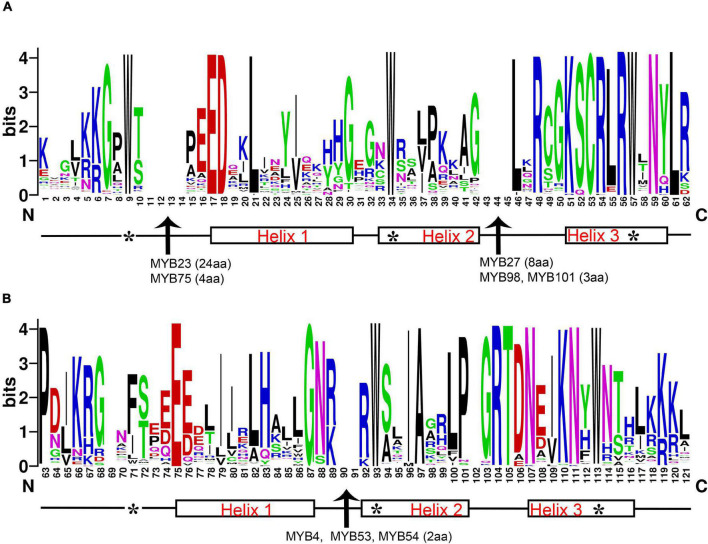
Characterization of R2 **(A)** and R3 **(B)** MYB repeats of longan R2R3-MYB proteins. The position score indicated the degree of conservation of each amino acid at the specific position within sequences. The positions of the three α-helices are marked (helices 1–3). Arrows represent amino acid insertion sites detected in the R2 repeat of genes *DlMYB23*, *DlMYB27*, *DlMYB75*, and *DlMYB98* and the R3 repeat of *DlMYB53*, *DlMYB54*, and *DlMYB93*. The length of each insertion (amino acid, aa) is highlighted in parentheses. Characteristic tryptophan (W) residues are marked with the symbol “*”.

The 119 R2R3-MYB amino acid sequence logos of R2 and R3 repeats were produced to show the sequence characterization and frequency of the most widespread amino acids at each position (Figure 1 and Supplementary Figure 1). In general, the R2 and R3 repeats of MYB domains had ∼108 basic residues (including the linker region). Critical amino acid insertions were detected in both R repeats, especially in the Helix2 (H2) segment of R2 and R3. The R2R3-MYB domains showed amino acid insertion points for DlMYB23 (24aa), DlMYB27 (8aa), DlMYB98 (3aa), and DlMYB101 (3aa) in R2, DlMYB4 (2aa), DlMYB53 (2aa), and DlMYB54 (2aa) in R3 (Figure 1 and Supplementary Figure 1). The results confirmed the conservation of the MYB domain, but amino acid compositions and lengths of the regions outside the MYB domain were highly variable.

A series of highly conserved and regularly distributed Trp (W) residues were detected in R2 and R3 repeats of DlMYBs, which are noted for landmarks of plant MYB proteins. Interestingly, only the second and third Trp residues were found to be conserved in the R3 repeat and the first Trp was generally replaced by a hydrophobic amino acid, such as Phe (F) or Ile (I). Apart from Phe and Ile, the substitution with the amino acids Met (M), Leu (L), and Try (Y) were also found in the first Trp residue in the R3 repeat at Trp-62 position of DlMYB proteins (DlMYB6, DlMYB10, DlMYB51, DlMYB57, DlMYB72, DlMYB81, DlMYB82, DlMYB98, DlMYB101, and DlMYB114). In addition, the Trp residues, Cys-45, Arg-48 in the R2 repeat, Leu-53 in the linker region, and Glu-66, Gly-78 in the R3 repeat were conserved in all DlMYB proteins (Figure 1 and Supplementary Figure 1). These highly conserved amino acid residues were mainly distributed in the third helix and the helix-turn-helix (HTH) motif. In DlMYB32 and DlMYB75, the Pro-55 was replaced with Ala and Ser in the R3 repeat, respectively, implying they may have different evolutionary divergence rates.

### Phylogenetic Analysis and Classification of Longan R2R3-MYB Proteins

**FIGURE 2:**
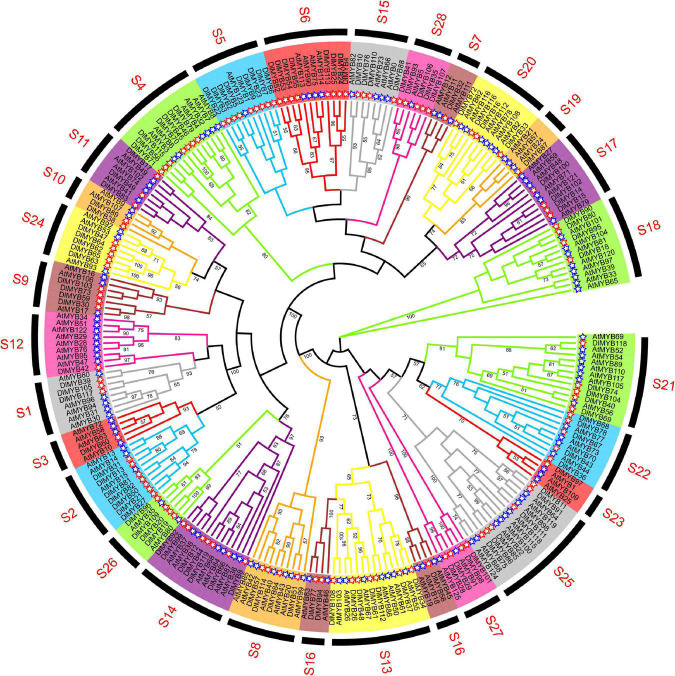
Phylogenetic analysis of longan and *Arabidopsis* MYB proteins. The full-length amino acid sequences of longan (119) and Arabidopsis (126) MYB proteins were compared using RAxML software to generate ML phylogenetic trees with 1,000 bootstrap values. The Evolview3 was used to visualize and display > 50 bootstrap values at the nodes. Red and blue stars represent DlMYB and AtMYB proteins, respectively. All MYB proteins were divided into 28 subgroups (S1–S28).

The maximum likelihood (ML) phylogenetic tree of 119 DlMYB and 126 AtMYB proteins showed that all R2R3-MYBs were categorized into 28 subgroups (designed as S1–S28) based on sequence similarity and topology (Figure 2). Each clade in the ML tree contained R2R3-MYB proteins from longan and Arabidopsis. Five AtMYBs and thirteen DlMYBs that could not be retrieved in the previously constructed AtMYB phylogenetic tree were rearranged in this study, in S26–S28, according to the phylogenetic analysis of Arabidopsis and longan MYBs. Eventually, all 119 longan R2R3-MYB proteins were divided into 28 subgroups, including S1 (three members), S2 (seven members), S3 (one member), S4 (seven members), S5 (nine members), S6 (eight members), S7 (one member), S8 (four members), S9 (four members), S10 (two members), S11 (three members), S12 (one member), S13 (seven members), S14 (seven members), S15 (four members), S16 (three members), S17 (four members), S18 (four members), S19 (one member), S20 (four members), S21 (five members), S22 (five members), S23 (one member), S24 (five members), S25 (six members), S26 (four members), S27 (four members), and S28 (five members) (Figure 2 and Supplementary Table 1). Not only the gene duplications but gene loss events seem to have an impact on the expansion and contraction of MYB members after the divergence of longan and Arabidopsis genomes. As shown in Figure 2, some subgroups (S2, S5, S6, S24, S27, S28) of the phylogenetic tree harbored twice as many longan MYB members as Arabidopsis MYBs. The opposite was also observed in subgroups 7, 10, 12, 16, 19, and 23, where the number of DlMYB proteins was less than half that of AtMYB proteins.

### Motif Composition, Gene Structure, and Promoter *Cis*-Acting Element Analysis

**FIGURE 3:**
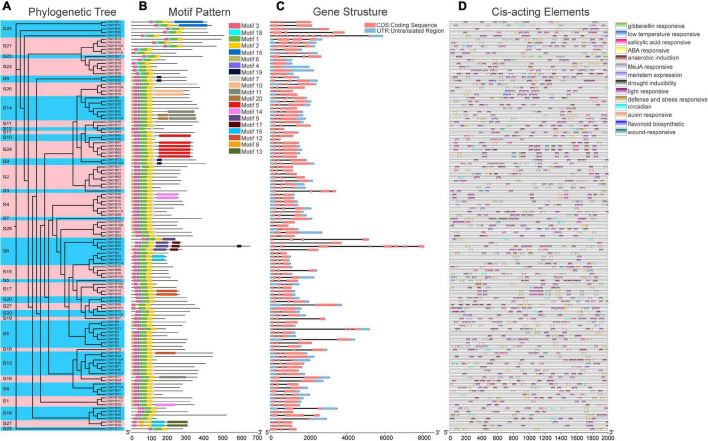
Phylogenetic tree, conserved motifs, gene structural, and *cis*-acting element analysis of DlMYBs. **(A)** An Maximum Likelihood (ML) tree of 119 DlMYBs proteins with 1,000 bootstrap replicates. **(B)** The conserved motifs in DlMYBs were predicted using the MEME Suite web server. **(C)** Exon/intron structures of the longan *R2R3-MYB* genes. The introns, exons, and UTRs are indicated by orange boxes, black lines, and blue boxes, respectively. **(D)** The *cis*-acting elements of the longan R2R3MYB proteins were predicted using PlantCARE.

The ML phylogenetic tree constructed for 119 DlMYB proteinsalone also revealed that all DlMYB proteins were divided into 28 subgroups (Figure 3A). In order to have a more comprehensive understanding of the diversity and conserved domains of these *R2R3-MYB* genes in longan, conserved motifs of these DlMYB protein sequences were examined. A total of 20 conserved motifs were examined and designated as motif 1 to 20 (Figure 3B, Supplementary Figure 2, and Supplementary Table 4). The majority of R2R3-MYB protein sequences had four significantly similar conserved motifs and motif orders. Ninety-seven out of 119 R2R3-MYB protein sequences contained motif 3, motif 4, motif 1, and motif 2 present in that order, and these motifs were composed of 21, 11, 40, and 29 amino acids, respectively (Figure 3B and Supplementary Table 4). Those four motifs were found to encode the MYB DNA-binding domain. Motifs 3, 4, and the left part of motif 1 were composed of the R2 repeat, and the right part of motif 1 and motif 2 were found in the R3 repeat. In other 17 R2R3-MYB proteins, motif 4 in the R2 repeat was replaced by motif 18. In addition, motif 4 and motif 1 of DlMYB28 and DlMYB29 were replaced by motif 8. Three R2R3-MYB proteins, DlMYB98, DlMYB100, and DlMYB101 only contained motif 1, motif 2, and motif 3. Almost all DlMYB proteins (118/119) contained motif 2 and motif 3 with the exception of DlMYB 42 that did not have motif 2. Most DlMYB proteins from the same clade had a similar motif composition. For instance, motifs 9 and 17 were shared by the members in S6, motifs 8 and 13 were exclusively in S27, and motif 15 was unique to S25, suggesting that these unique motifs may contribute to the differentiation in specific functions.

The exon/intron organization was analyzed to investigate the structural diversity of *DlR2R3-MYB* genes. The number of introns in longan *R2R3-MYB* genes ranged from zero to thirteen (Figure 3C). Most (111/119) of the coding sequences of *R2R3-MYB* genes in longan were disrupted by one to three introns, with the exception of six *DlMYB* genes (*DlMYB36*, *DlMYB43*, *DlMYB67*, *DlMYB68*, *DlMYB78*, and *DlMYB101*) that completely lacked introns, indicating the conserved intron number in the *R2R3-MYB* genes. Only two *DlMYB* genes had an excess of introns, including *DlMYB75* (13 introns) and *DlMYB55* (8 introns). Most genes clustered in the same subgroup exhibited highly similar gene structures and intron numbers, which could strongly support the accuracy of the classification of subgroups.

The predicted results for *cis*-regulatory elements showed that a total of fourteen types of *cis*-regulatory elements were predicted in *DlR2R3-MYB* genes. The most widespread *cis*-acting elements in the promoter region of the *DlMYB* genes were light responsiveness (119/119), anaerobic induction (102/119), and ABA responsiveness elements (101/119) (Figure 3D and Supplementary Tables 5, 6), while the least common ones were wound-responsive (4/119), flavonoid biosynthetic genes regulation (13/119) and circadian (23/119). Moreover, the promoters of 79 (66.39%), 70 (58.82%), and 56 (47.06%) *DlMYB* genes were predicted to contain *cis*-acting elements associated with MeJA (methyl jasmonate), GA (gibberellin), and SA (salicylic acid), respectively. The detailed information regarding the *cis*-regulatory elements in longan *DlMYB* genes is given in Supplementary Tables 5, 6. All 14 types of *cis*-acting elements could be divided into three major functional categories, including cellular development or photoresponsive elements (two types of elements), phytohormone (five types of elements), and environmental stress (seven types of elements). In general, *DlR2R3-MYB* gene promoters containing environmental stress-related elements (398) were the most common followed by phytohormone-related ones (358).

### Chromosomal Distribution, Duplication Events and Interspecies Synteny Analysis

**FIGURE 4:**
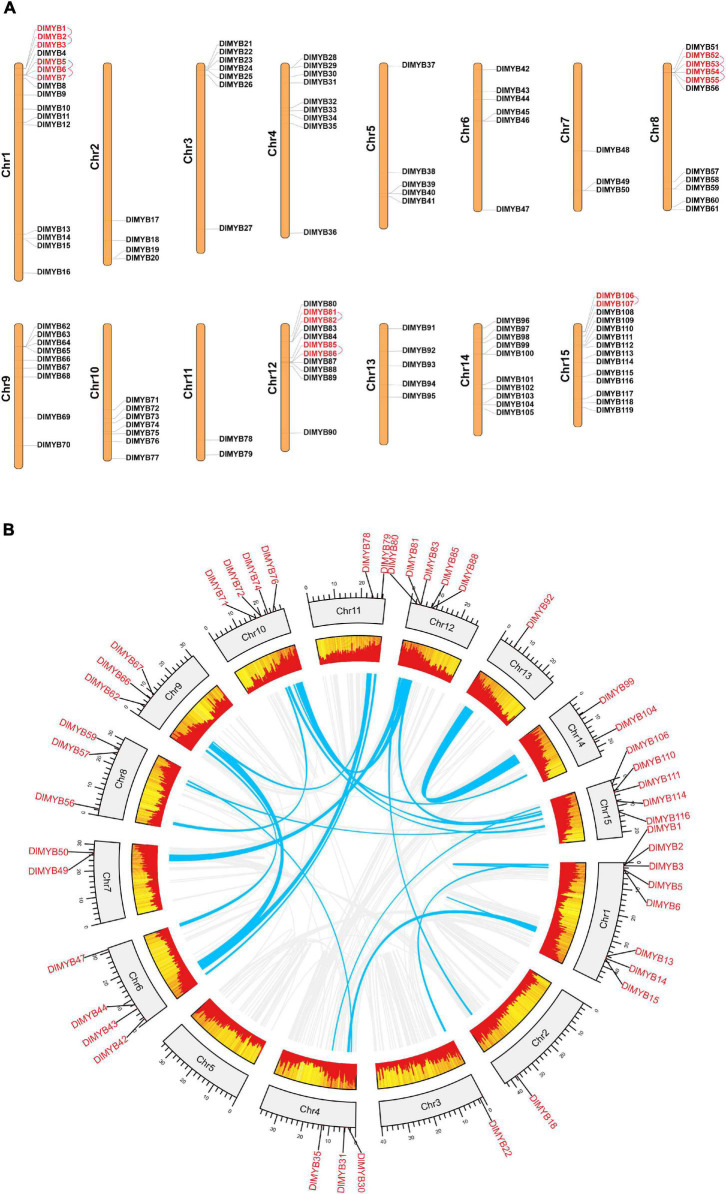
Chromosomal location, tandem duplication, and segmental gene duplication analysis of the *DlMYB* genes. **(A)** The distribution map of *DlMYB* genes on the 15 chromosomes of longan (genes with tandem clusters are marked in red). **(B)** Circos plot of *DlMYB* genes in the longan genome. Gene pairs with segmental duplications (SDs) are indicated by blue lines, and gene density was highlighted by red rectangular boxes.

Genome chromosomal location analysis showed that 119 *R2R3-MYB* genes were unevenly distributed on 15 chromosomes (Figure 4A), and each chromosome had at least two *DlR2R3-MYB* genes. DlChr1 harbored the largest group of *R2R3-MYB* genes (16/119), followed by DlChr15 with 14 *R2R3-MYB* genes and DlChr12 with 11 genes, while only two genes were located on DlChr11. *DlR2R3-MYB* genes tend to cluster to the proximal end of some chromosomes, including DlChr1, DlChr2, DlChr3, DlChr4, DlChr8, DlChr10, and DlChr12.

In our study, a total of 25 segmental duplications (SD) involving 44 *MYB* genes were identified on different chromosomes (Figure 4B and Supplementary Tables 7, 8). *DlMYB* genes were located within synteny blocks on almost all chromosomes except for DlChr5. The SD events occurred mostly on chromosomes 1, 12, and 15 (Supplementary Table 8). Some *DlR2R3-MYB* genes, such as *DlMYB2*, *DlMYB78*, and *DlMYB110*, were involved in multiple duplications. In total, six tandem duplications (TD) clusters consisting of 16 *DlR2R3-MYB* genes were identified in DlChr1, DlChr8, DlChr12, and DlChr15 (Figure 4A). The distribution of TD genes on chromosomes were consistent with aforementioned chromosomes with a high-density of *MYB* genes. These results indicated that approximately 43.7% of *DlMYB* genes might be generated by duplication events, which played a vital role in the expansion of the *MYB* gene family in longan (Supplementary Table 7). The *Ka*/*Ks* values (non-synonymous/synonymous substitution ratios) were calculated to understand the selection mode of segmentally and tandemly duplicated *R2R3-MYB* gene pairs in longan. *Ka* had a range of values from 0.06 to 0.52 while *Ks* values varied from 0.14 to 4.36 (Supplementary Table 8). In particular, one duplicated gene pair *DlMYB14-DlMYB15* had no *Ka* and *Ks* value (NaN). Through manual checking, we found that mutations between *DlMYB14* and *DlMYB15* sequences occurred at the nucleic acid level but their amino acid sequences remained unchanged. The *Ka*/*Ks* ratios of 35 *DlMYB* gene duplicated pairs (including 10 tandem and 25 segmental duplication gene pairs) ranged from 0.05 to 0.84 (Supplementary Table 8) (all less than 1), indicating that longan *R2R3-MYB* duplicated genes had undergone negative purifying selection in the process of evolution and contribute largely to the maintenance of function in the longan *R2R3-MY*B gene family. The average *Ka*/*Ks* value of tandem duplication genes was 0.34, two-fold that of segmented duplication genes (0.17) (Supplementary Figure 6). Furthermore, the *Ka*/*Ks* value of each orthologous *DlMYB* gene pair was also calculated in the *Sapindaceae* family (Supplementary Figure 6 and Supplementary Table 9). Nearly all of the 289 (289/291) orthologous gene pairs between longan and litchi, longan and yellow horn had Ka/Ks values of < 1, except for two gene pairs *DlMYB32*-*LITCHI026531.m1* (Ka/Ks = 1.20) and *DlMYB100*-*LITCHI018670.m1* (Ka/Ks = 1.78) between longan and litchi, implying that purifying selection may be the dominant force driving the evolution of *R2R3-MYB* genes in the lineage of *Sapindaceae*. The Ka/Ks mean values of the *R2R3-MYB* syntenic gene pairs between longan and Arabidopsis, longan and litchi, longan and yellow horn orthologs were 0.15, 0.32, and 0.23, respectively (Supplementary Figure 6 and Supplementary Table 9).

**FIGURE 5:**
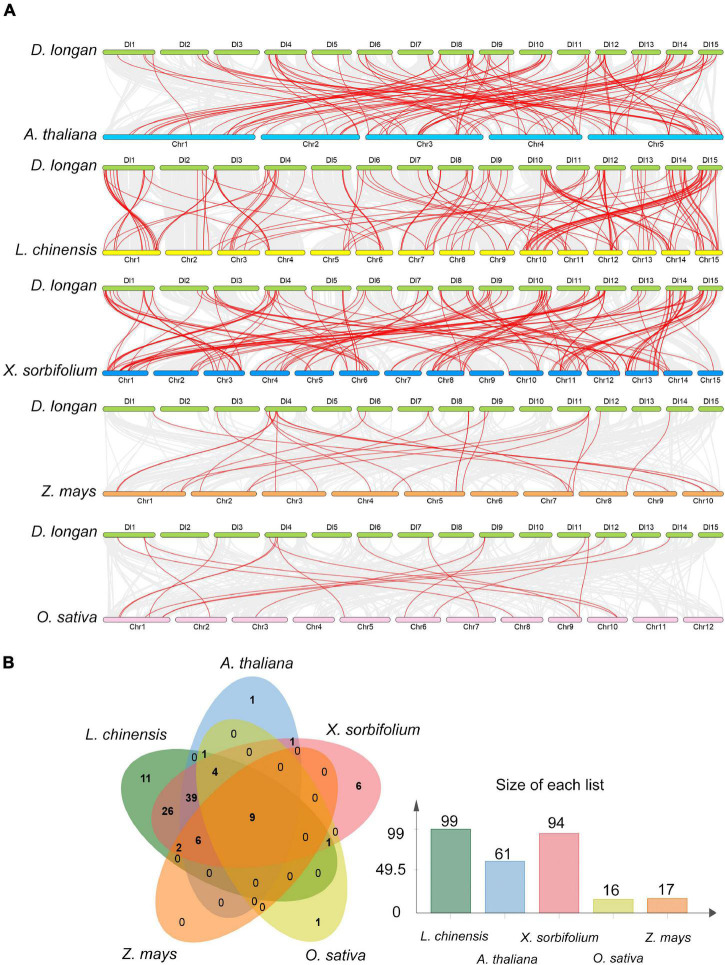
Collinearity analysis of the *DlMYB* genes. **(A)** Synteny relationship of *R2R3MYB* genes between longan and five other representative plant species: Arabidopsis, litchi, yellow horn, maize, and rice. Gray lines in the background indicate the collinear blocks within two genomes, and red lines highlight the syntenic MYB gene pairs. **(B)** The *R2R3MYB* genes formed the colinear pairs between longan and the other five species. Bar graphs indicate the number of genes in colinear pairs.

To gain more insight into the evolutionary model of the longan *R2R3-MYB* gene family, the comparative syntenic maps of longan along with three representative dicots including Arabidopsis, litchi (*Litchi chinensis*) and yellow horn (*Xanthoceras sorbifolium* Bunge), and two monocots, rice (*Oryza sativa* L.) and maize (*Zea mays*) were generated (Figure 5A). Litchi and yellow horn were phylogenetically closer to longan, which were also in the family of *Sapindaceae*. The results revealed that 99 *DlMYB* genes were involved in the formation of a syntenic relationship with those in litchi, followed by yellow horn (94 genes involved), Arabidopsis (61), maize (17), and rice (16) (Figure 5B). The maximum number of ortholog *DlR2R3-MYB* gene pairs were found in *DlMYB*-*XsMYB* (148) and *DlMYB*-*LcMYB* (143) followed by *DlMYB*-*AtMYB* (102), while the minimum number were found in *DlMYB*-*ZmMYB* (25) and *DlMYB*-*OsMYB* (17) (Supplementary Table 9), which is consistent with their taxonomic positions. Notably, a total of nine *DlMYB* genes were predicted to form collinear pairs with genes from all the other five species (Supplementary Table 10). Eighty-five syntenic gene pairs were only found between longan and other dicots (Figure 5B). For example, the gene pair *DlMYB38*-*AT3G02940.1* was only detected between longan and Arabidopsis, *DlMYB51*-*EVM0018212.1*-*AT1G66230.1* was only found among longan, Arabidopsis, and yellow horn. A total of 26 MYB genes were only found in longan, litchi, and yellow horn (Supplementary Table 10). Additionally, 18 *AtMYB* genes were predicted to be paired with at least two longan genes (Supplementary Table 9), suggesting the newly evolved functions of redundant *DlMYB* genes. The detailed information of syntenic gene pairs is provided in Supplementary Tables 9, 10.

### Subcellular Localization of DlMYB Proteins

**FIGURE 6:**
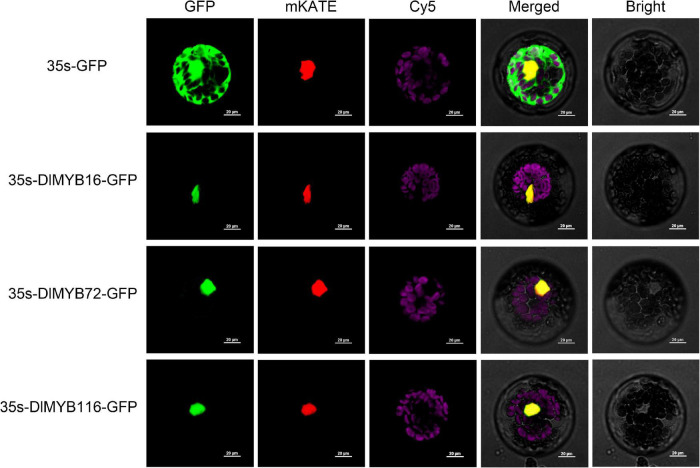
Nuclear localization of DlMYB16, DlMYB72, and DlMYB116 proteins in Arabidopsis protoplasts. GFP: green fluorescent protein; mKATE: nuclear marker; Cy5: chloroplast; Bright: bright field; merged: combined mKATE and GFP and bright field. Bars, 20 μm.

Subcellular localization predictions revealed that all 119 DlMYB proteins were located in the nucleus (Supplementary Figure 7 and Supplementary Tables 1, 3). To experimentally validate the predicted subcellular localization, we selected three potential flowering-associated *DlMYB* genes (*DlMYB16*, *DlMYB72*, and *DlMYB116*) for analysis in Arabidopsis protoplasts. These three genes were expressed at significantly high levels in flower or flower bud tissues compared with other tissues. The NLS-mKATE and Cy5 were applied in each transformed design as a marker for nuclear and chloroplast localization, respectively. The results showed that DlMYB16, DlMYB72, and DlMYB116 proteins were localized to the nucleus (Figure 6), which was consistent with subcellular localization predictions.

### Expression Profile of *DlMYB* Genes in Different Tissues and Gene Ontology Annotation Analysis

**FIGURE 7:**
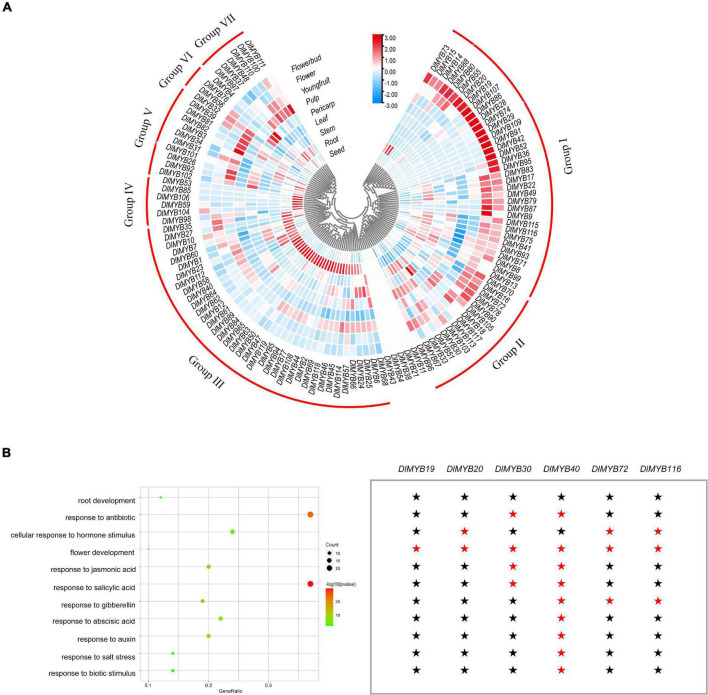
Tissue-specific expression and GO enrichment analysis of DlMYB genes. **(A)** The expression levels of *DlMYB* genes in nine tissues were classified into groups I–VII. Different colors indicate log2 transformed expression levels, with red indicating high expression levels and blue indicating low expression levels. **(B)** Some *DlMYB* genes were classified as related to stress response, development, and hormones. The size of the circle indicates the number of *DlMYB* genes, and the color gradient indicates the size of the *P*-value. Six *DlMYB* genes are categorized into “flower development” function. A red pentagram represents that the gene is relevant, while a black pentagram indicates that the gene is not involved in the category.

The differential expression patterns of *DlR2R3-MYB* genes in nine tissues were analyzed using RNA-seq data (Figure 7A, Supplementary Figures 3, 4, and Supplementary Tables 11, 12). The results revealed that the *DlR2R3-MYB* genes showed varying expression patterns in different tissues and most genes displayed tissue-specific expressions. In general, 119 *R2R3-MYB* genes were clustered into seven groups according to the preferential expression tissues, which were designated as group I to group VII (Figure 7A). Group I genes were highly expressed in flowers and flower buds. Group II genes were widely expressed in different tissues, which indicated that they were involved in various organ formation processes in longan. Group III genes were mainly expressed in the roots, suggesting that they might execute certain functions in the root development. Genes in group IV-VII exhibited preferential expression in seeds, young fruits, pericarps, and pulps, respectively. In total, 74 (62.18%), 58 (48.74%), 47 (39.50%), 62 (52.10%), 57 (47.90%), 69 (57.98%), 30 (25.21%), 62 (52.10%), 50 (42.02%) *DlR2R3-MYB* genes were expressed (TMM > 1) in roots, stems, leaves, flower buds, flowers, young fruits, pulps, pericarps and seeds, respectively (Supplementary Figure 3 and Supplementary Table 12). Flowers and flower buds were observed to have the most *DlR2R3-MYB* genes that had TMM ≥ 100, with 10.08 and 7.56%, respectively, suggesting that more *R2R3-MYB* genes were highly expressed in flower tissues than in other tissues. Of 119 *DlMYB* genes, 98 genes (82.35%) were expressed in at least one tissue (Supplementary Table 11). By contrast, five genes (*DlMYB11*, *DlMYB21*, *DlMYB38*, *DlMYB43*, and *DlMYB54*) in longan were not expressed (TMM = 0) in any of the examined tissues, possibly because they could only be expressed under given conditions, or detected in certain tissues that were not examined in our study, or they were pseudogenes. Three *DlMYB* genes (*DlMYB67*, *DlMYB70*, and *DlMYB79*) were observed to have high expression levels (TMM > 10) in all nine tissues examined. It is worth noting that fourteen genes (*DlMYB8*, *DlMYB16*, *DlMYB17*, *DlMYB33*, *DlMYB35*, *DlMYB67*, *DlMYB70*, *DlMYB72*, *DlMYB78*, *DlMYB79*, *DlMYB87*, *DlMYB93*, *DlMYB115*, and *DlMYB116*) showed extremely high expression levels (TMM > 100) in flowers and flower buds. Some genes exhibited flower-specific expressions. Four genes (*DlMYB28*, *DlMYB29*, *DlMYB42*, and *DlMYB109*) were expressed exclusively in flower buds, and *DlMYB87* was expressed only in flowers and flower buds.

A total of 153 GO items were enriched by the GO annotation with a cut-off value of *P* ≤ 0.05, among which 146 GO terms were classified into the biological process category and only seven into the molecular function category (Supplementary Table 13). Fifty-eight of all *DlMYB* genes (48.74%) were assigned to categories related to hormones, stress response, and tissue development (Figure 7B). Interestingly, six DlMYB genes (*DlMYB19*, *DlMYB20*, *DlMYB30*, *DlMYB40*, *DlMYB72*, and *DlMYB116*) were assigned to the “flower development” category. Among them, *DIMYB19* belonged exclusively to the “flower development” category and *DIMYB20* was only associated with two categories “flower development” and “cellular response to hormone stimulus,” while *DlMYB40* was also related to other several categories such as development and salt stress response (Figure 7B and Supplementary Table 13). Regarding their expression levels, however, *DlMYB40* was lowly expressed in all examined tissues except for roots and stems. *DIMYB19* and *DIMYB20* showed slightly higher expression (with TMM of 10∼12) in flower buds. Two genes, *DlMYB72* and *DlMYB116*, that had extremely high expression levels in flowers or flower buds were given more attention in the following analysis.

### Expression Pattern of DlMYBs in Off-Season FI Induction by KClO_3_

**FIGURE 8:**
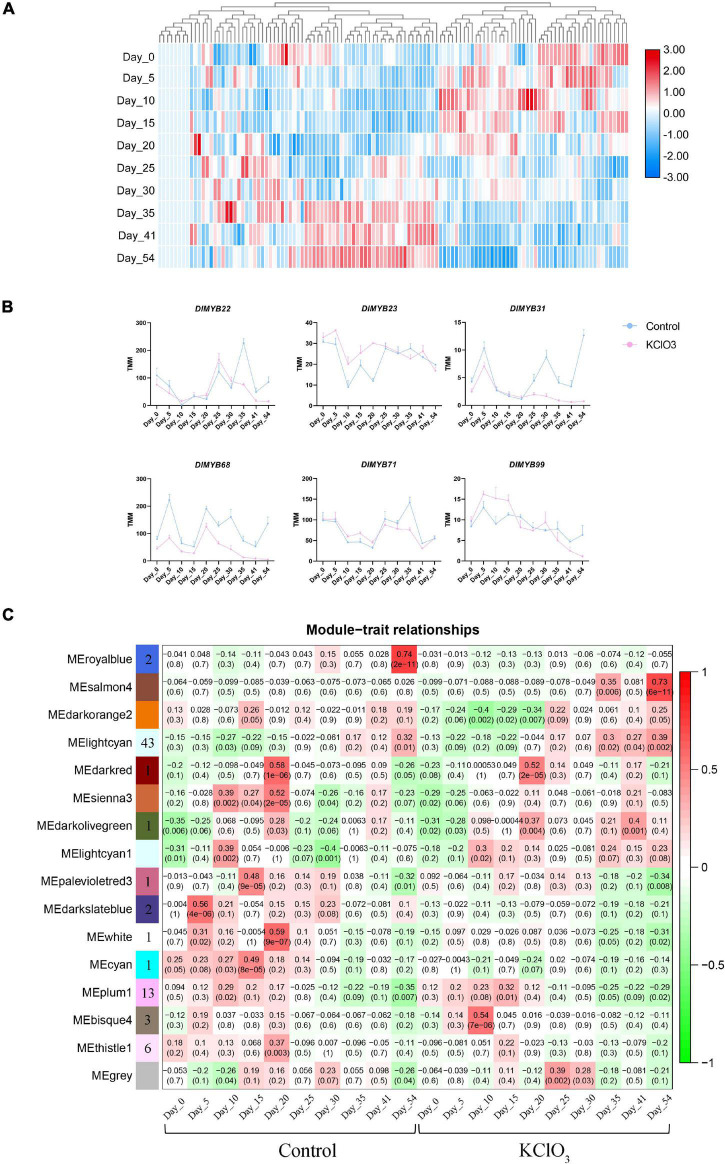
Expression patterns of DlMYB genes under KClO_3_ treatment. **(A)** The heat map displays the hierarchical clustering of the 119 *DlMYB* genes at different time points, with red indicating high expression levels and blue indicating low expression. **(B)** The line graphs display the effect of KClO_3_ treatment on the expression of *DlMYB* genes. The blue line represents the control and the pink line represents KClO_3_ treatment. **(C)** The heatmap from WGCNA of differentially expressed genes (DEGs) in off-season flower induction at different treatments and developmental stages. Each row corresponds to one module resulting from an WGCNA eigengene value. Each column corresponds to the control treatment and the KClO_3_ treatment at different time-points. The color and number of each cell at the row-column intersection indicate the correlation coefficient between one specific module and one time point under control or KClO_3_ treatment. Darker red represents a higher degree of correlation. The number in brackets indicate the significance (*p*-value) of the correlation coefficient. The number of *DlMYB* genes in each module is shown in the leftmost column.

To further investigate the biological function of *DlMYB* genes responding to KClO_3_ treatment, ten time points with untreated control and KClO_3_ treatment were used to detect the expression profiles of *DlMYB* genes in apical buds. The hierarchical cluster analysis was performed based on the expression (TMM values) of *DlMYB* genes treated with KClO_3_ at different periods (Figure 8A and Supplementary Table 14). In the TMM analysis results, 20.17% (24/119) of all *DlMYB* genes were observed to have no expression (TMM < 1) at different time points (Supplementary Table 14). A total of 18 *DlMYB* genes (15.13%) were highly expressed (TMM > 10) at all ten time points, among which, *DlMYB35*, *DlMYB93*, *DlMYB98*, and *DlMYB101* were extremely highly expressed (TMM > 100). The time course analysis showed that six genes were significantly upregulated or downregulated (*Padj* < 0.05) under KClO_3_ treatments at different time points compared with control. For instance, the gene expression of *DlMYB31* and *DlMYB68* were inhibited after KClO_3_ treatment compared to the control, indicating they were negatively regulated by KClO_3_ (Figure 8B and Supplementary Table 14). *DlMYB22* and *DlMYB71* were inhibited in the later stages after KClO_3_ treatment. In contrast, the expression of *DlMYB23* and *DlMYB99* were promoted in the initial stages by the KClO_3_ treatment. In addition, Some genes in the same subfamilies tended to exhibit similar expression patterns. For example, *DlMYB33* and *DlMYB116* in subfamily 20 were up-regulated at day 5 and day 10, and then commenced to decline at later time points (Figure 2 and Supplementary Table 14).

To elucidate the regulatory pathway of off-season flowering among *DlMYBs* and other genes, coexpression networks were generated incorporating 16 modules via WGCNA. The heatmap of ten time points under control and KClO_3_ treatment showed that 74 differentially expressed *R2R3-MYB* genes were distributed in eleven modules represented by different colors (MEbisque4, MEcyan, MEdarkolivegreen, MEdarkred, MEdarkslateblue, MElightcyan1, MEpalevioletred3, MEplum1, MEroyalblue, MEthistle1, and MEwhite) (Figure 8C). After filtering out the low-interacting genes based on the weight values reflecting the connectivity between genes in each module (the thresholds of weight values were shown in Supplementary Table 15), 35 *R2R3-MYB* genes remained that potentially interact with 567 genes. KEGG pathway enrichment analysis revealed that the “plant hormone signal transduction” pathway (ko04075) was enriched with 18 genes (Supplementary Figure 9A), and GO enrichment showed that these genes were mainly associated with “DNA binding TF activity” (Supplementary Figure 9B).

Among the eleven distinct modules, the MElightcyan module with 211 genes had an expression pattern correlated with the late period of KClO_3_ (35-54 DAT) (Figure 8C and Supplementary Table 15). A gene expression network was developed to further mine candidate *DlMYB* genes (Supplementary Figure 8). Fifteen *DlMYB* genes were contained in the 211 genes. *DlMYB113* was identified as hub genes, with several TF genes such as *HISTONE H2A 11* (*HTA11, Dil.04g007090.1*), *EARLY IN SHORT DAYS 7/TILTED 1* (*TIL1, Dil.13g008690.1*), and *MITOTIC ARREST-DEFICIENT 2* (*MAD2*, *Dil.10g018750.1*). The MEplum1 module was found to be correlated with the early stage (15 DAT) of KClO_3_ treatment. In total, 133 genes were involved in this module, including *DlMYB30*, *DlMYB41* and *DlMYB60*, and other genes such as *TEOSINTE BRANCHED1/CYCLOIDEA/PROLIFERATING CELL FACTOR1-20* (*TCP20, Dil.12g008890.1*) and *HISTIDINE-CONTAINING PHOSPHOTRANSMITTER 2* (*AHP2, Dil.04g009050.1*). The MEdarkolivegreen module with 7 genes were associated with the middle stage (20 DAT) of KClO_3_ treatment. *DlMYB13* was identified as a hub gene in the central location, closely linked to *HUMAN WDR5 HOMOLOG A* (*WDR5a, Dil.04g005430.1*). The MEbisque4 module was comprised of 58 genes that were specifically and highly associated with the early period (10 DAT) of KClO_3_ treatment. This module included three R2R3-MYB TF genes (*DlMYB8*, *DlMYB33*, and *DlMYB78*). *DlMYB*8 was identified as the top hub gene and was directly connected to *WRKY75* and *ERF114*.

### Validation of *DlMYB* Genes Expression at Different Tissues

**FIGURE 9:**
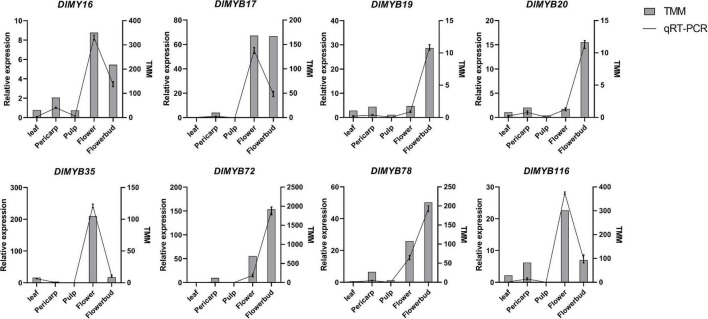
qRT-PCR and RNA-seq analyses of eight selected DlMYB genes. The samples from flowers, flower buds, leaves, pulps, and pericarps show a range of expression changes of selected *DlMYB* genes. The left and right sides of the *Y*-axis indicate relative expression levels and TMM, respectively. To confirm the tissue-specific expression of the *DlMYB* genes, the expression pattern of selected *DlMYB* genes was calculated as a fold relative to the expression level of the pericarp.

To validate the expression of *DlMYB* genes in flower development, eight representative *DlMYB* genes (*DlMYB16*, *DlMYB17*, *DlMYB19*, *DlMYB20*, *DlMYB35*, *DlMYB72*, *DlMYB78*, and *DlMYB116*) were selected for qRT-PCR analysis (Figure 9). Overall, the trends of the qRT-PCRs for the eight *DlMYB* genes were consistent with the results of RNA-seq analysis. However, a slight difference existed due to differences between longan cultivars used, the cultivation conditions of plants, and the geographical location of tissue collection.

As shown in Figure 9, *DlMYB17* and *DlMYB72* were observed to be expressed in the pericarps, flowers, and flower buds specifically. *DlMYB16* and *DlMYB116* were detected to have similar expression patterns and high expression in flowers and flower buds, suggesting a potential redundancy in their functions. Similar trends were found for *DlMYB19* and *DlMYB20* in subfamily 26. In addition, *DlMYB35* was specifically expressed in leaves, flowers, and flower buds. Strongly in line with our theorized expectations, the eight selected genes were significantly highly expressed in both flowers and flower buds compared with other tissues in longan.

## Discussion

The MYB gene family constitutes one of the largest TFs implicated in the regulation of a wide array of physiological processes in plants. Thus far, this present study is the first to conduct *in silico* systematic and genome-wide characterization of the *R2R3-MYB* gene family in the longan genome and reveal their potential roles in longan flower development.

### Evolutionary Tracing of *DlMYB* Genes

In this study, a total of 119 R2R3-MYB proteins were identified in the “Shixia” longan genome, which were classified into 28 subgroups (Figure 2 and Supplementary Table 1). A considerable fraction of subgroups (S1–S25) in our classification were consistent with the subgroupings in other plants (Dubos et al., 2010). Division of R2R3-MYBs into subgroups based on conserved sequence motifs would identify clades whose members commonly exhibited similar biological functions (Millard et al., 2019a). The numbers of *R2R3-MYB* genes in longan and Arabidopsis were remarkedly different in some subgroups within the phylogenetic tree (Figure 2). This phenomenon was also observed in many other species such as potato (Sun et al., 2019), maize (Du et al., 2012a), grape (Wong et al., 2016), and flax (Tombuloglu, 2020). For one reason, the number of *R2R3-MYB* genes may be related to the quality of the assembled genome. The marked improvement of the chromosome-level longan genome assembly would expand and enrich the predicted *DlMYB* gene pool. In comparison with our result, only 35 (Zheng et al., 2020) and 98 (Lin et al., 2017) *DlMYB* genes were identified based on transcriptome data and the previous scaffold-level longan assembly using Illumina data, respectively. For another, the variation in the total number of *R2R3-MYB* genes and subgroup genes among plants may have arisen from evolutionary divergence during which the expansion/contraction of *MYB* genes occurred in different species (Jiang and Rao, 2020). TD, SD and whole genome duplication (WGD) events have long been known to occur throughout plant evolution and perform a significant role in new gene generation and gene family expansion (Van de Peer, 2004). The results showed that tandem duplication and segmental duplication events were unevenly distributed throughout all chromosomes, with 16 *DlMYB* genes identified as TDs and nearly three times more *DlMYB* genes (44) classified as SDs (Figure 4 and Supplementary Tables 7, 8). Similar trends of more SDs were observed in Arabidopsis (Cannon et al., 2004) and potato (Sun et al., 2019), suggesting a more crucial role of SD events in the expansion of *MYB* genes compared to TD events. More SD events and fewer TDs have commonly been present in gene families involved in housekeeping or core regulatory functions (Cannon et al., 2004). *Ka*/*Ks* was used to reflect the selection pressure on plants during evolution. The *Ka*/*Ks* ratios of all 35 tandemly and segmentally duplicated *DlR2R3-MYB* gene pairs were less than 1 (Supplementary Table 8), indicating that they might have been subject to purifying selection with constraint functional divergence after species-specific duplication events. This result corresponds well with previous observations in many plant species (Abbas et al., 2021; Wang et al., 2021). Although R2R3-MYB TFs contain “non-MYB regions” that are highly variable, disordered and compositionally biased, they have been evolving at a neutral rate and under the pressure of purifying selection with evolutionary constraints (Millard et al., 2019a).

Interspecies synteny analysis demonstrated that *DlR2R3-MYB* genes had higher collinearity with *R2R3-MYB* genes of three dicot species, especially Litchi, and yellow horn in the same family of *Sapindaceae* (Figures 5A,B and Supplementary Tables 9, 10), suggesting their divergence from the common dicot ancestor. In addition, some syntenic gene pairs were only present between longan and other dicots. For instance, 39 syntenic gene pairs were identified between longan and all examined dicots. Those pairs may appear after the divergence of dicotyledon and monocotyledon species. There were 26 syntenic *DlMYB* genes exclusively found in litchi and yellow horn, implying those genes may be *Sapindaceae* specific *MYB* genes, which have arisen after the divergence of *Sapindaceae* and *Brassicaceae*. Eighteen *AtMYB* genes paired with at least two longan genes indicate that additional longan genes would be generated due to duplication events and play vital roles during the evolution of longan. Nine *DlMYB* genes were identified to have synteny with their orthologs in five plants analyzed, suggesting that these genes may have existed prior to the divergence of these plant species and are highly conserved in evolution (Figure 5B and Supplementary Tables 10, 11).

### Structural Characteristics and Functional Prediction of *DlMYB* Genes

We constructed a separate ML phylogenetic tree of 119 DlMYB proteins, which showed that either tandem replication or segmental replication gene pairs were classified into the same subgroup, except for the *DlMYB42*-*DlMYB79* segmental replication gene pair (Figure 3A). In addition, the motif composition and the distribution of exon/intron structures varied among *DlMYB* genes (Figures 3B,C). For example, *DlMYB98*, *DlMYB100*, and DlMYB101 contained only three motifs, while 16 *DlMYB* genes harbored the largest number of 7 motifs (Figure 3B). In general, a high degree of conservation in the MYB structural domain (R2 and R3 repeats) were found and members of the same subgroup tended to have similar motif combinations. The motifs that made up R2 and R3 repetitive sequences were highly conserved and mainly distributed at the N terminus, while specific motifs were present at the C terminus (Figure 1 and Supplementary Figure 1). The motifs 9 and 17 belonged exclusively to S6, motifs 8 and 13 were only found in S27, and motif 15 was unique in S25. Previous researches revealed that unique motifs may be associated with the functional divergence of DlMYBs (Millard et al., 2019b). Gene structure analysis showed that members in the same subgroup were also inclined to have similar exon/intron structures and intron numbers. Four genes in subgroup 6, *DlMYB52*, *DlMYB53*, *DlMYB54*, and *DlMYB55*, however, showed significantly different gene structures from each other although they formed a tandem gene cluster in Chr8. Consistent with previous studies (Abbas et al., 2021), most *R2R3-MYB* genes were found to contain only two introns with the exception of *DlMYB75* and *DlMYB55* that contained 13 and 8 introns, respectively. Introns were found to have functions in promoting cellular resistance to starvation by enhancing the repression of ribosomal protein genes (Parenteau et al., 2019).

The *cis*-acting element analysis results showed that DlMYB proteins were predicted to have various elements related to phytohormone, environmental stress, and cellular development (Figure 3D and Supplementary Tables 5, 6). Phytohormones such as ABA, ethylene, GA, and MeJA are involved in the regulation of plant flower development and senescence (Bao et al., 2020; Huang et al., 2021). The promoters of the *DlMYB* genes were predicted to contain several *cis*-acting elements associated with hormone responses, implying the potential role of *DlMYB* genes in the regulation of flower development. GO enrichment analysis also indicated that some DlMYB proteins have functions in response to hormones, tissue development and environmental stress (Figure 7B and Supplementary Table 13). Among them, the “flower development” pathway was enriched with six genes, indicating their potential functions in flowering (Figure 7B and Supplementary Table 13). In agreement with previous research results in Arabidopsis (Dubos et al., 2010), the MYB binding sites involved in flavonoid biosynthetic regulation (MBSI) elements were found in promoters of thirteen longan *R2R3-MYB* genes. Another 61 *DlMYB* genes contained the drought inducibility element known for its role in response to drought stress, suggesting that more than half of the *DlR2R3-MYB* genes might be involved in the drought stress response.

### Expression Patterns and Potential Functions of *DlMYB* Genes Involved in Flowering

Many R2R3-MYB members are involved in regulating plant development (Millar and Gubler, 2005; Cheng et al., 2009; Phan et al., 2011; Song et al., 2011; Huang H. et al., 2017). The expression analysis allowed us to further estimate the functions of *DlMYB* genes in longan. Our results revealed that varying expression patterns were observed among longan tissues and most genes exhibited tissue-preferential expression (Figure 7A), suggesting their tissue-specific roles in the development of differentiated tissues. Five genes were not expressed in all examined tissues, which were probably pseudogenes or could only be detected in other tissues that we did not collect. The functions of *DlMYB* genes were closely linked to their orthologous counterparts in Arabidopsis. Four longan genes, *DlMYB18*, *DlMYB80*, *DlMYB81*, and *DlMYB90* clustered with *AtMYB33* and *AtMYB65* in subgroup 18, which could promote anther and pollen development in Arabidopsis (Millar and Gubler, 2005). In addition, *DlMYB18* and *DlMYB80* formed segmental replication gene pairs in the longan genome and had synteny with *AtMYB97*/*AtMYB101*/*AtMYB120* that were found to be synergistic pollen tube factors and could control the fertilization process (Liang et al., 2013). These four longan *MYB* genes abovementioned were found to be active in flowers or young fruits, suggesting their potential roles in pollen development. Similarly, *DlMYB19*, *DlMYB20, DlMYB96*, and *DlMYB109*, which grouped with *AtMYB80* in subgroup 26, also showed high expression in flowers, indicating they may participate in flower development, especially in regulating pollen development (Phan et al., 2011).

Moreover, *AtMYB21* and *AtMYB24* that take part in the regulation of anther development (Cheng et al., 2009; Song et al., 2011) grouped with *DlMYB87* in subgroup 19. *DlMYB87* was extremely highly expressed in flowers, suggesting its possible involvement in regulating anther development. *AtMYB108* in subfamily 20 can regulate stamen maturation in Arabidopsis (Mandaokar and Browse, 2009). Another gene *AtMYB62* in S20 is a regulator of phosphorus stress in Arabidopsis. Overexpression of *AtMYB62* inhibits the synthesis of gibberellic acid (GA) and the expression of the flowering regulators *SCO1* and *SUPERMAN*, ultimately leading to delayed flowering in Arabidopsis (Devaiah et al., 2009). Their homologs in longan, *DlMYB16*, *DlMYB33*, *DlMYB72*, and *DlMYB116* were observed to have high expression in flowers. Among them, *DlMYB72* and *DlMYB116* had similar expression patterns as segmental replication genes and formed syntenic gene pairs with *AtMYB62*/*AtMYB116* and *AtMYB62*, respectively (Figure 7A and Supplementary Tables 10, 11). Therefore, these genes may have similar functional characteristics in the regulation of flower development. Beyond its function in flowering, *AtMYB108* also participated in the regulation of the leaf senescence process, acting by binding to a specific region of *ANAC003* promoter to form a MYB-NAC regulatory complex (Chou et al., 2018). Its homologs *DlMYB16* and *DlMYB116* were also highly expressed in leaves, suggesting that genes in the same subgroup may play different biological functions. In subgroup 14, *AtMYB68* was demonstrated to specifically regulate the root growth (Jiang et al., 2004), and its homologs *DlMYB8*, *DlMYB12*, *DlMYB44*, *DlMYB66*, *DlMYB99*, and *DlMYB119* were also found to be highly expressed in the roots, presumably having an important role in longan root development. The high levels of association between longan MYB expression patterns and the roles of their orthologs in Arabidopsis may be evidence of functional conservation between homologous R2R3-MYB members of Arabidopsis and longan.

The qRT-PCR experiments demonstrated the high expression levels of *DlMYB19*, *DlMYB20*, *DlMYB72*, and *DlMYB116* in flowers and flower buds, which were also annotated in the “flower development” pathway by GO enrichment analysis, further suggesting that these genes may play an active part in the regulation of flower development (Figure 9).

### Potential Functions of *DlMYB* Genes Related to Stress and Metabolite Synthesis

Multiple R2R3MYB proteins were found to be capable of abiotic and biotic stress tolerance in plants (Dubos et al., 2010). *AtMYB15*, which clustered with the segmental replication gene pairs *DlMYB13* and *DlMYB31* in subgroup 2, was demonstrated to possess cold stress tolerance and drought resistance (Ding et al., 2009; Kim et al., 2017). *AtMYB15* enhances the susceptibility to freezing by negatively regulating *CBF* genes which are essential for freezing resistance in Arabidopsis, whereas MPK6-mediated phosphorylation reduces the affinity of *AtMYB15* to bind to the *CBF3* promoter to enhance cold stress tolerance (Kim et al., 2017). Meanwhile, the overexpression of *AtMYB15* enhanced ABA-mediated drought resistance and salt tolerance in Arabidopsis (Ding et al., 2009). Interestingly, *DlMYB13* and *DlMYB31* happened to have low-temperature responsiveness (LTR) elements and several ABA-responsive (ABRE) elements, and *DlMYB31* was assigned to the “response to cold” pathway in GO enrichment analysis (Supplementary Tables 5, 6, 9–13), indicating their potential role in frost and drought resistance. Also, lignin biosynthesis is regulated by *AtMYB15*, which plays a key role in regulating plant growth and development as well as in plant innate immunity by promoting the synthesis of secondary cell walls (Kim et al., 2020). These two homologous *DlMYB* genes may also have similar biological functions that remain to be uncovered. In subgroup 22, five longan genes (*DlMYB36*, *DlMYB43*, *DlMYB67*, *DlMYB68*, and *DlMYB78*) formed a single cluster with four orthologous *AtMYB* genes (*AtMYB44*, *AtMYB70*, *AtMYB73*, and *AtMYB77*), which have been reported to be involved in the abiotic stress response (Jung et al., 2008; Zhao et al., 2014; Yang et al., 2020). For example, *AtMYB44* among them functions in the regulation of ABA-mediated stomatal closure as well as drought tolerance (Jung et al., 2008). The *cis*-acting element prediction showed that these four longan genes all have drought inducibility elements except for *DlMYB68* (Supplementary Tables 5, 6). In particularly, *DlMYB78* contained four drought inducibility elements, indicating it may have greater potential in response to drought induction. In terms of transcript expression, *DlMYB67* was extremely highly expressed in all tissues. qRT-PCR experiments showed that *DlMYB78* was extremely highly expressed in flowers and flower buds, which may promote flowering under drought induction (Figure 9 and Supplementary Tables 9–11).

R2R3-MYB proteins are involved in different primary and secondary metabolic regulations in plants (Dubos et al., 2010). In Arabidopsis subgroup5, *AtMYB123* ensures a high accumulation of proanthocyanidin (PAs) by forming the MYB-BHLH-WDR (MBW) complex (Baudry et al., 2004). The AtMYB123 homolog, *DlMYB7*, was highly expressed in roots, stems, leaves, flowers and young fruits, with a possible function in proanthocyanidin synthesis (Figure 7A and Supplementary Figure 4). Four *AtMYB* genes, *AtMYB75*/*PAP1*, *AtMYB90*/*PAP2*, *AtMYB113*, and *AtMYB114* have been reported to control anthocyanin biosynthesis (Teng et al., 2005; Gonzalez et al., 2008), which formed a subgroup (S6) with the tandem replication gene cluster *DlMYB52*/*DlMYB53*/*DlMYB54*/*DlMYB55* in longan (Figures 2, 4A). *DlMYB32*, *AtMYB11*/*PFG2*, *ATMYB12*/*PFG1*, and *AtMYB111*/*PFG3* were classified as subgroup 7. The *AtMYB*s in this group mainly participate in the regulation of flavonol biosynthesis (Dubos et al., 2010). Previous studies have found *AtMYB21* and *AtMYB24* in subgroup 19, that cluster with *DlMYB87*, played conserved regulatory roles in flavonol biosynthesis in Arabidopsis anthers and pollen by activating the *flavonol synthase 1* (*AtFLS 1*) gene (Stracke et al., 2010; Shan et al., 2020). The flower-specific expressed gene *DlMYB42* clustered with *AtMYB28* (*HAG1*), *AtMYB29* (*HAG2*), and *AtMYB86* (*HAG2*) with functions involved in aliphatic glucosinolate biosynthesis (Gigolashvili et al., 2008) in subgroup 12.

### Expression of *DlMYB* Genes in Off-Season Induced Flowering by KClO_3_

The transcriptome dynamics of 119 *DlMYB* genes in apical buds at different periods under KClO_3_ treatment were analyzed(Figure 8A and Supplementary Table 14). The time course analysis of 119 *DlMYB* genes at different periods under control and KClO_3_ treatment promoted the expression of *DlMYB23* and *DlMYB99* at the initial stage, which may be activated in response to the KClO_3_ induction (Figure 8B and Supplementary Table 14). Compared with the gene expression under control without KClO_3_ treatment, we observed that the expression of four genes (*DlMYB22*, *DlMYB31*, *DlMYB68*, and *DlMYB71*) were inhibited under KClO_3_ treatment, indicating that they may have a negative effect on flowering by KClO_3_ treatment. In general, *DlMYB* genes in the same subfamily tended to have similar expression patterns.

Co-expression network analysis via WGCNA was used to identify the role of *DlMYB* genes in KClO_3_-mediated FI (Figure 8C). Based on the FLOR-ID of Arabidopsis (Bouché et al., 2016), we found seven flower-related genes out of these 567 genes, including *HTA11*, *WDR5a*, *TIL1*, *SENSITIVE TO FREEZING 6* (*SFR6*), *HISTONE ACETYLTRANSFERASE OF THE MYST FAMILY 1* (*HAM1*), *CURLY LEAF* (*CLF*), and *CYCLING DOF FACTOR 3* (*CDF3*). Some *DlMYB* hub genes were closely linked to other TF genes. For example, three *DlMYB* genes (*DlMYB8*, *DlMYB33*, and *DlMYB78*) were directly linked to TF genes such as *WRKY75* and *ERF114* in the MEbisque4 module (Supplementary Figure 8). The *WRKY75* could positively regulate flowering in Arabidopsis by GA-mediated binding to the promoter of the *FT* gene (Zhang et al., 2018). *WRKY2* and *WRKY34* repressed *AtMYB97*, *AtMYB101*, and *AtMYB120* expression during male gametogenesis (Lei et al., 2018). *DlMYB8* and *WRKY75* were up-regulated in the early stages of KClO_3_ treatment and presumably they may positively coregulate *FT* gene expression and eventually induce flowering in longan. In the MEroyalblue module, on the contrary, *DlMYB31*, *DlMYB51* and *ERF1* were down-regulated after KClO_3_ treatment. In Arabidopsis, *ERF1* delayed flowering by repressing the expression of the *FT* gene (Chen et al., 2021). The downregulation of those genes under KClO_3_ may attenuate the repression of *FT* expression and ultimately promoted flowering. In the MEplum1 module, *DlMYB41*, *TCP20*, and *AHP2* were all up-regulated in the earlier stages after KClO_3_ treatment (Supplementary Figure 8 and Supplementary Table 14). In Arabidopsis, interestingly, *AHP2* functioned in the cytokinin pathway to promote female gametophyte development and *TCP20* promoted the absorption of nitrate (Guan et al., 2014; Liu et al., 2017). Chlorate was a nitrate analog that could be absorbed by the nitrate transporters (Borges et al., 2004). Thus, *DlMYB41* and *TCP20* may together facilitate the utilization of KClO_3_, which was subsequently reduced to chlorite and hypochlorite and directly induced the stress response. Meanwhile, *DlMYB41* and *AHP2* were interacted to stimulate CK-mediated signal transduction. Eventually, they acted together to influence the expression of flowering-related genes and induce flowering. Whether and how these *DlMYB* genes function in longan flowering induction and development requires further experiments to elucidate.

## Conclusion

In the current study, a systematic identification of R2R3-MYB gene family members in longan was carried out. We identified a total of 119 full-length *R2R3-MYB* genes in longan, which were further divided into 28 distinct subgroups by phylogenetic classification. A comprehensive bioinformatics analysis was performed to investigate the protein physicochemical properties, motif composition, gene structure, and *cis*-element prediction of the *DlMYB* genes. An uneven distribution of *DlMYB* genes on 15 chromosomes was observed with at least two genes per chromosome. The synteny analysis indicated segmental replication events were major contributors to the expansion of the *R2R3-MYB* gene family in longan. Comparative syntenic mapping among longan and other five representative species provided insights into the evolutionary relationship of orthologous MYB members and implied the presence of functional differentiation led by duplication events during evolution. Subcellular localization analysis revealed that the selected flowering-associated *DlMYB* genes mainly function in the nucleus as predicted. Expression profiling of *DlMYB* genes indicates their tissue-preferential expression pattern and KClO_3_-induced off-season flowering effect. GO enrichment analysis revealed that six *DlMYB* genes were assigned to the “flower development” category. The selected eight genes were further proved to be highly expressed in flowers and flower buds by qRT-PCR, indicating their possible involvement in the flower development process. Expression patterns in KClO_3_-mediated FI indicated two up-regulated and four down-regulated *DlR2R3-MYB* genes were involved. These results will open up untapped avenues for further functional analysis of the *R2R3-MYB* genes to elucidate their roles in longan FI and floral organ development.

## Data Availability Statement

The datasets presented in this study can be found in online repositories. The names of the repository/repositories and accession number(s) can be found in the article/Supplementary Material.

## Author Contributions

JF and JZ conceived the study and designed the experiments. QC, JF, XZ, YF, BW, SX, and KZ carried out the experiments and analyzed the data. QC and JF wrote the manuscript. All authors read and approved the final manuscript.

## Conflict of Interest

The authors declare that the research was conducted in the absence of any commercial or financial relationships that could be construed as a potential conflict of interest.

## Publisher’s Note

All claims expressed in this article are solely those of the authors and do not necessarily represent those of their affiliated organizations, or those of the publisher, the editors and the reviewers. Any product that may be evaluated in this article, or claim that may be made by its manufacturer, is not guaranteed or endorsed by the publisher.
