# Supplementary material for: Genome-Wide Identification and Expression Analysis of the R2R3-MYB Transcription Factor Family Revealed Their Potential Roles in the Flowering Process in Longan (Dimocarpus longan)
Source: Front Plant Sci. 2022 Mar 25;13:820439. doi: 10.3389/fpls.2022.820439 (PMC8990856; doi:10.3389/fpls.2022.820439)
Supplement: Supplementary file 1 [file Data_Sheet_1.docx]

Supplementary Material

## Supplementary Figures

**
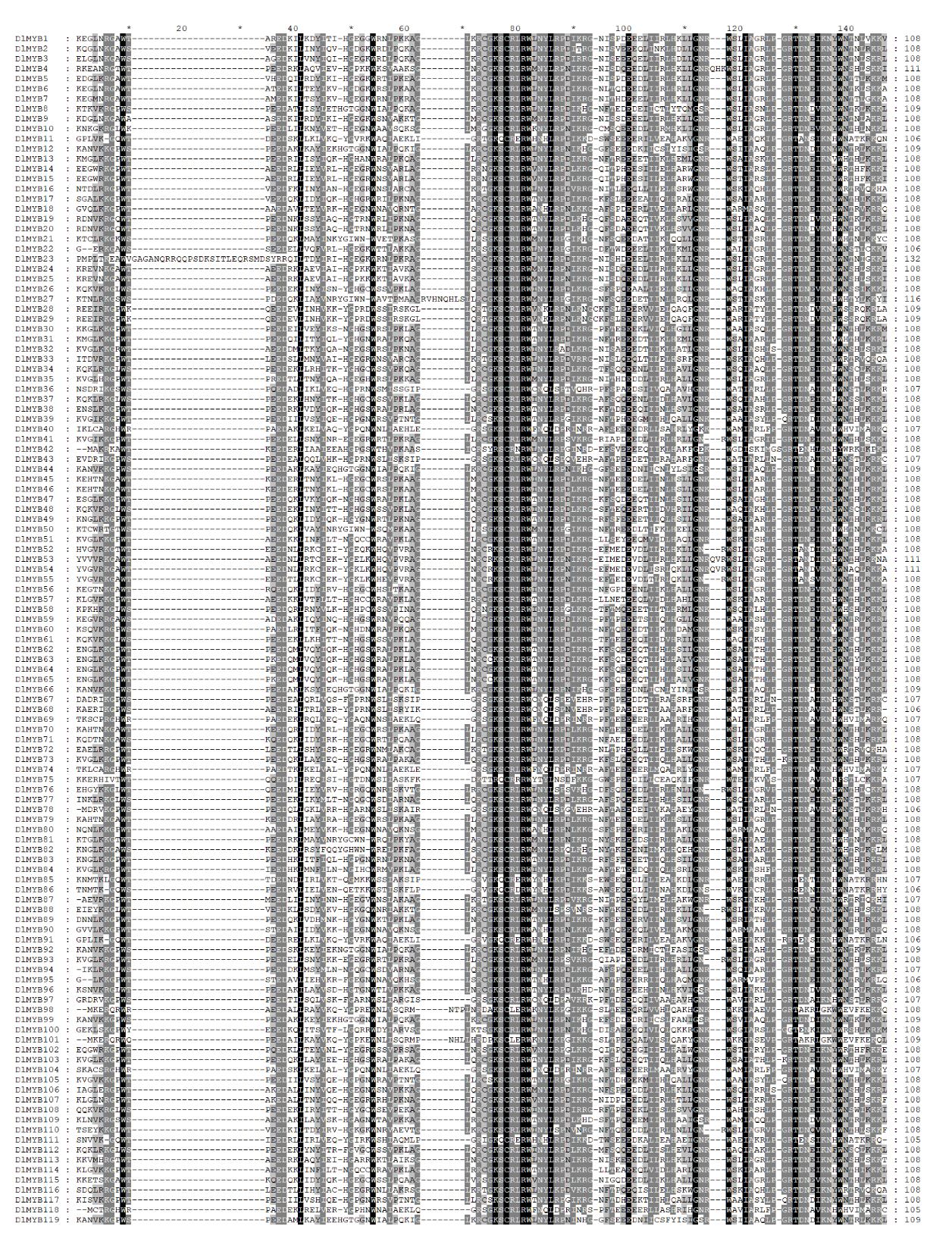
**

**Figure S1 Multiple sequence alignments of 119 DlMYB proteins.** The alignment was performed using ClustalX and visualized by GeneDoc.

**
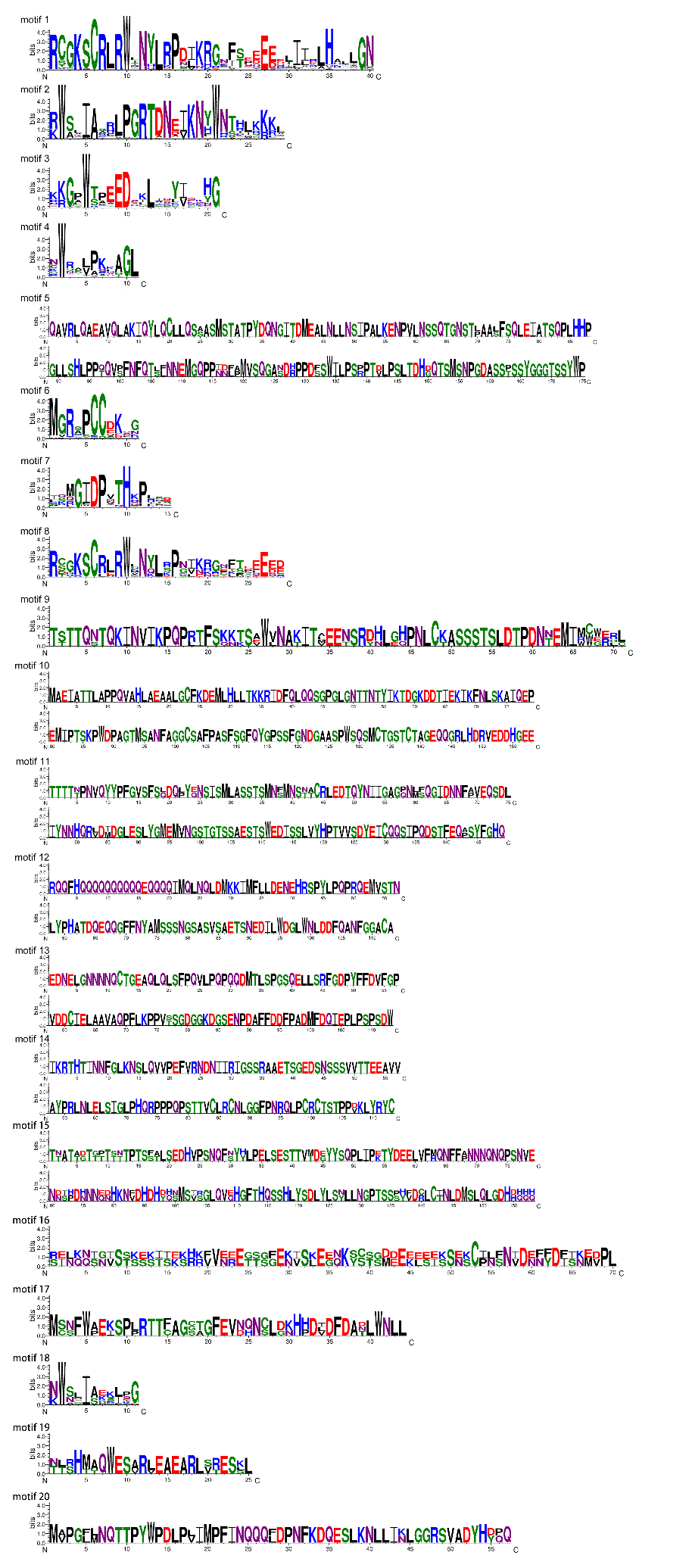
**

**Figure S2 The schematic representation of the 20 motifs in DlMYB proteins.**

**
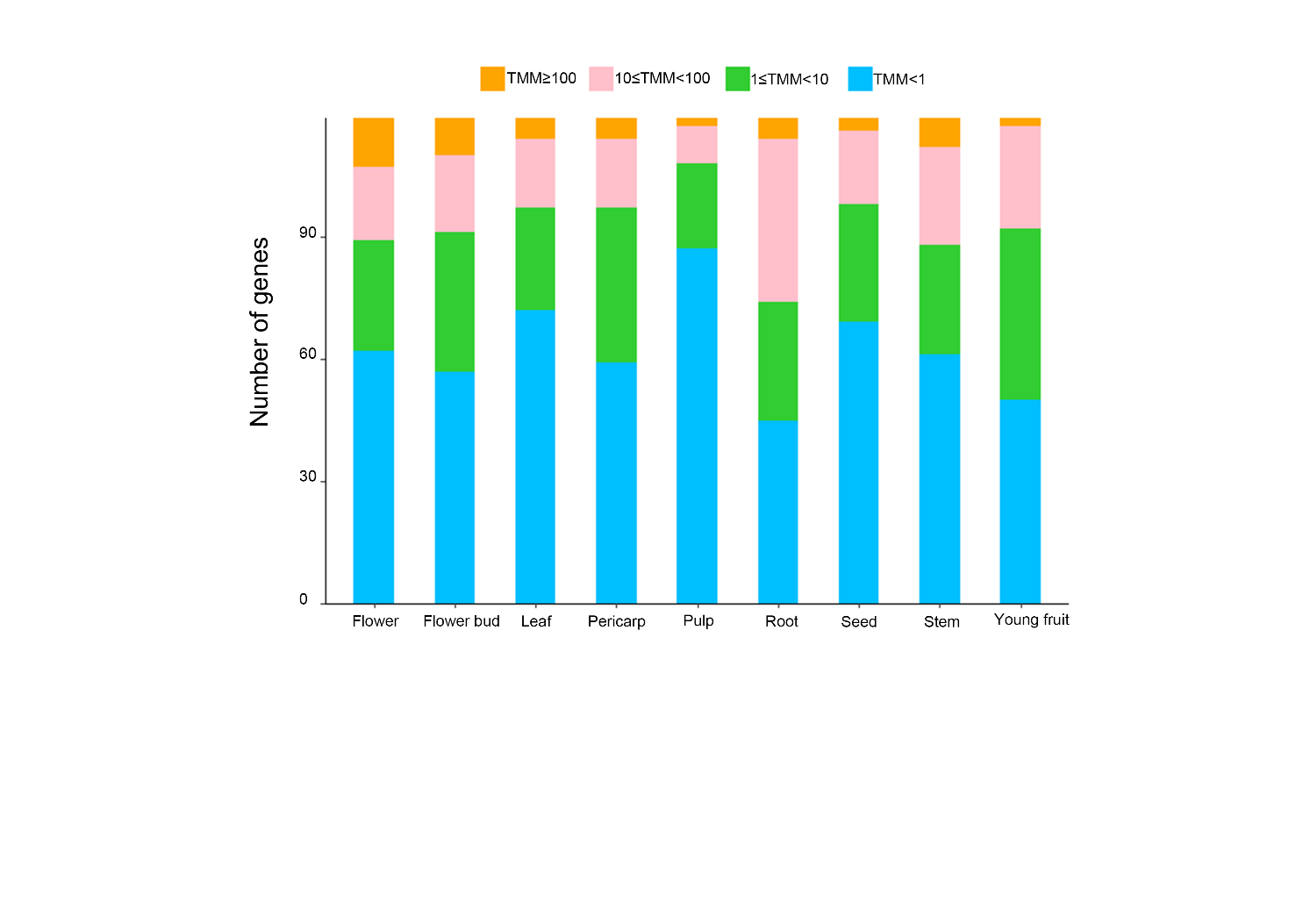
**

**Figure S3 The number of *DlMYB* genes expressed in nine tissues including flowers, flower buds, leaves, pericarps, pulps, roots, seeds, stems, and young fruits.**

**
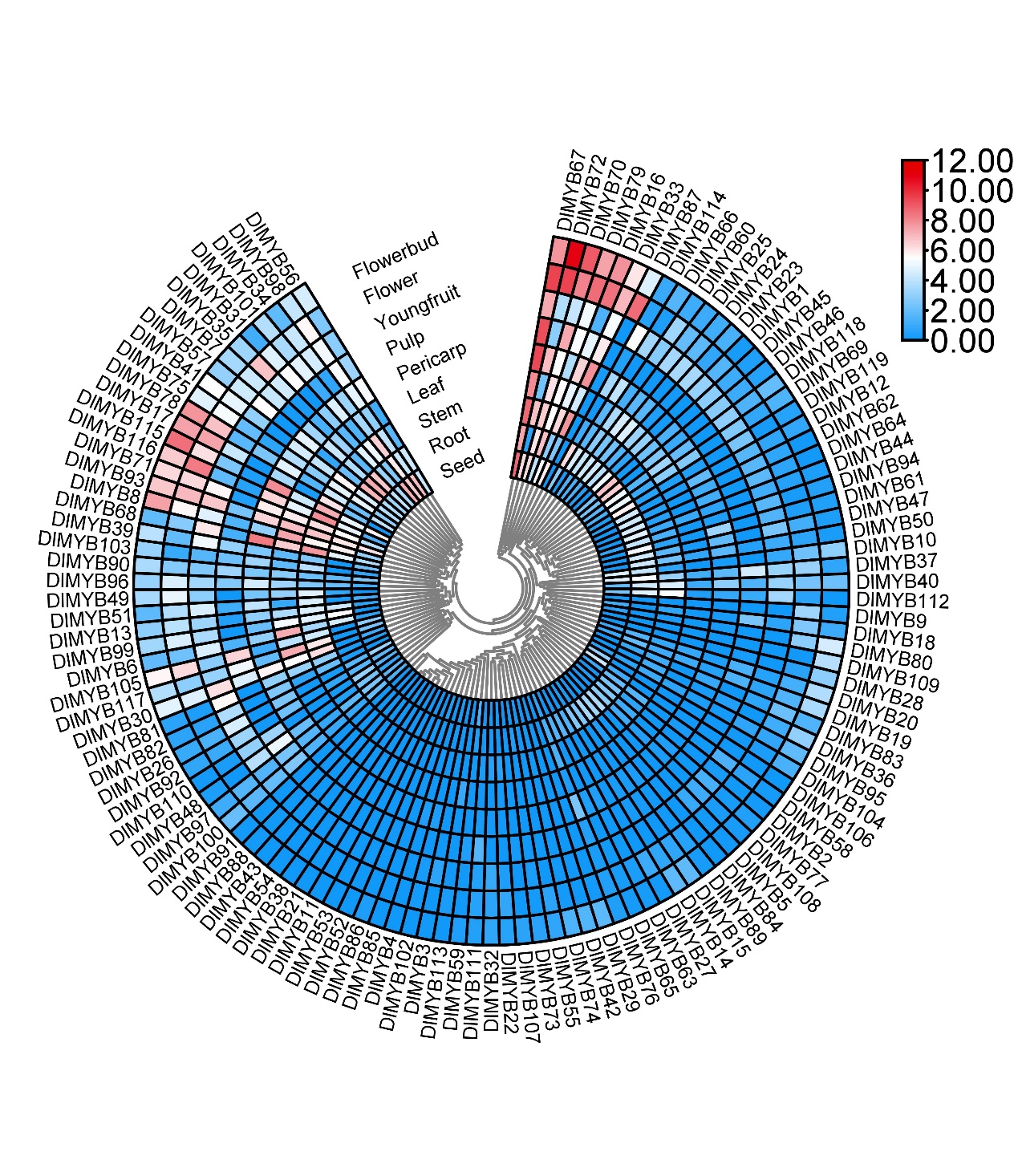
**

**Figure S4 Expression heat maps of *DlMYB* genes expressed in nine tissues of longan.** Expression values were normalized by log_2_(TMM + 1), with red indicating high expression levels and blue indicating low expression levels.

**
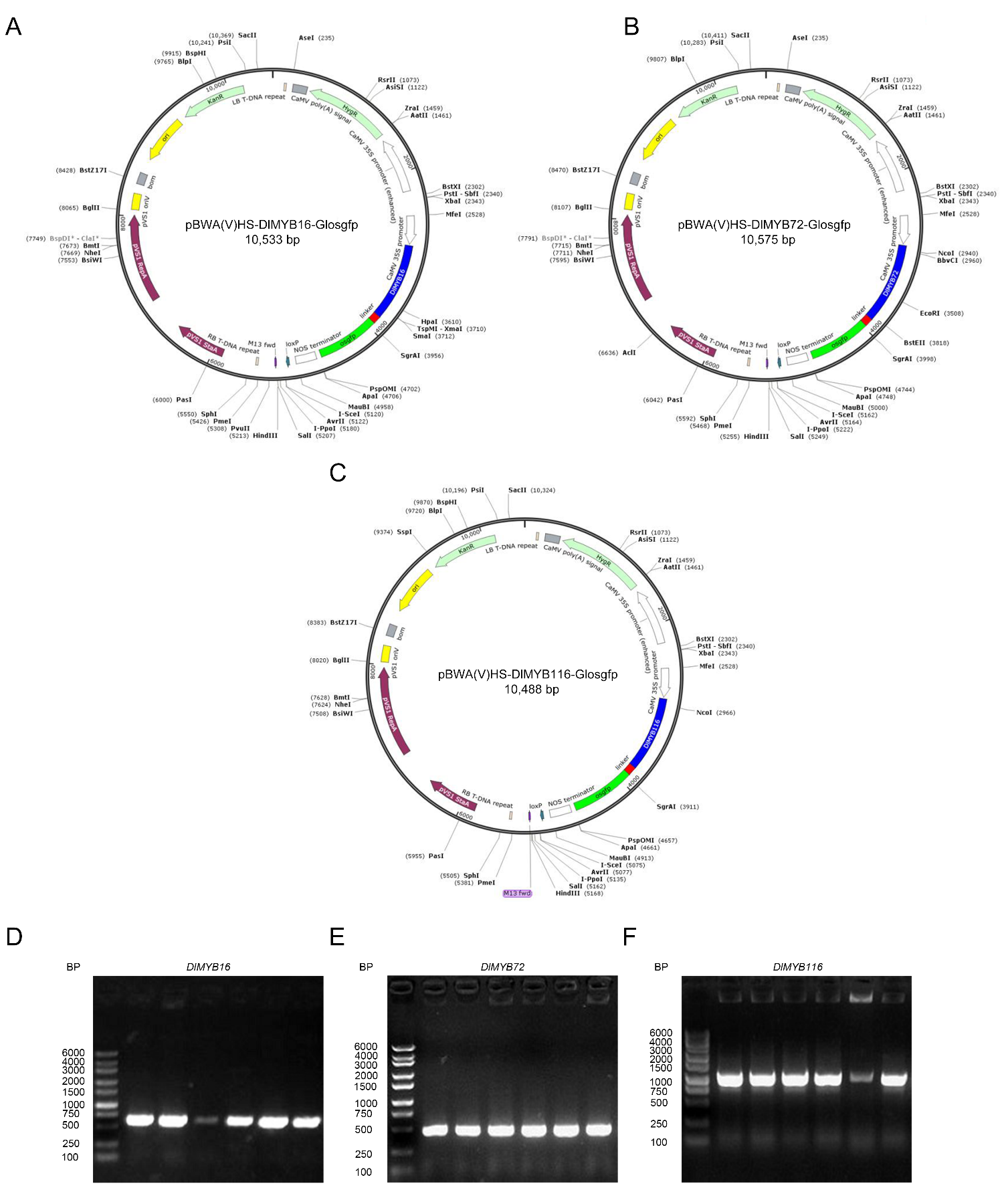
**

**Figure S5 The colony PCR identification and plasmid construction maps for subcellular localization. (A-C**) indicate the plasmid construction maps for *DlMYB16*, *DlMYB72*, and *DlMYB116* genes, respectively. Positions of three DlMYB genes were shown. Arrows indicate orientation of the respective genes. (**D-F**) indicate the colony PCR identification of pBWA(V)HS-DlMYB16-Glosgfp, pBWA(V)HS-DlMYB72-Glosgfp, and pBWA(V)HS-DlMYB116-Glosgfp vectors in *E. coli*, respectively. The bands on the left represent the DNA Marker: from top to bottom, 6000, 4000, 3000, 2000, 1500, 1000, 750, 500, 250, and 100 bp, respectively. The PCR bands on the right show that about 507 bp, 486 bp, and 1055 bp for *DlMYB16*, *DlMYB72*, and *DlMYB1116* were successfully amplified in *E. coli*, respectively.

**
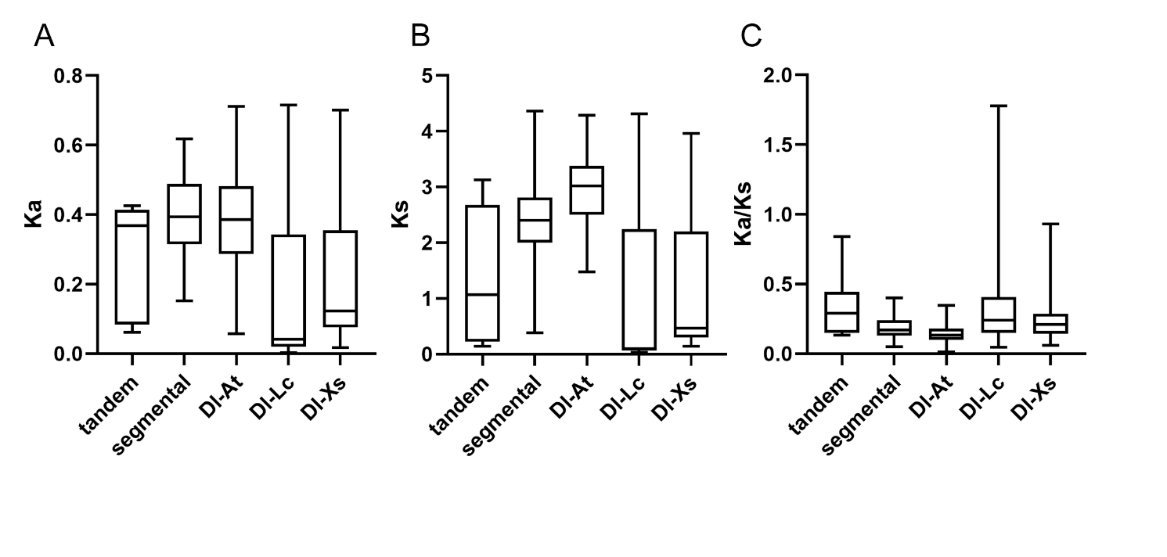
**

**Figure S6 The average values of Ka, Ks, and Ka/Ks of *R2R3-MYB* duplicated genes.** The horizontal axes in (A-C) represent *R2R3-MYB* tandem duplication (tandem), segmental duplication (segmental), and the collinear gene pairs between longan and Arabidopsis (Dl-At), litchi (Dl-Lc), and yellow horn (Dl-Xs), respectively.


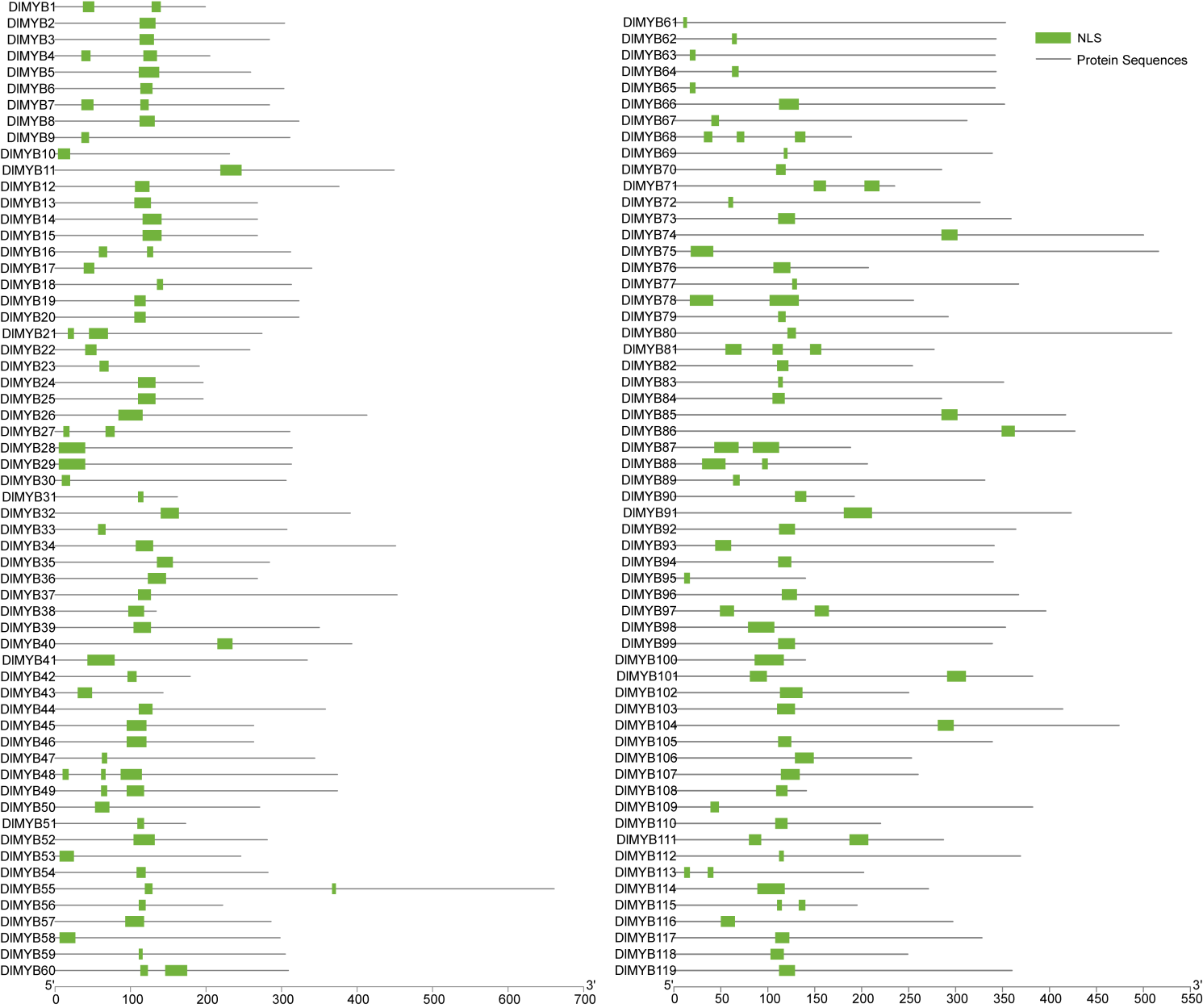


**Figure S7 The predicted nuclear localization signal (NLSs) for 119 DlMYBs.** The green rectangular boxes indicate the positions of predicted NLS sequences.

**
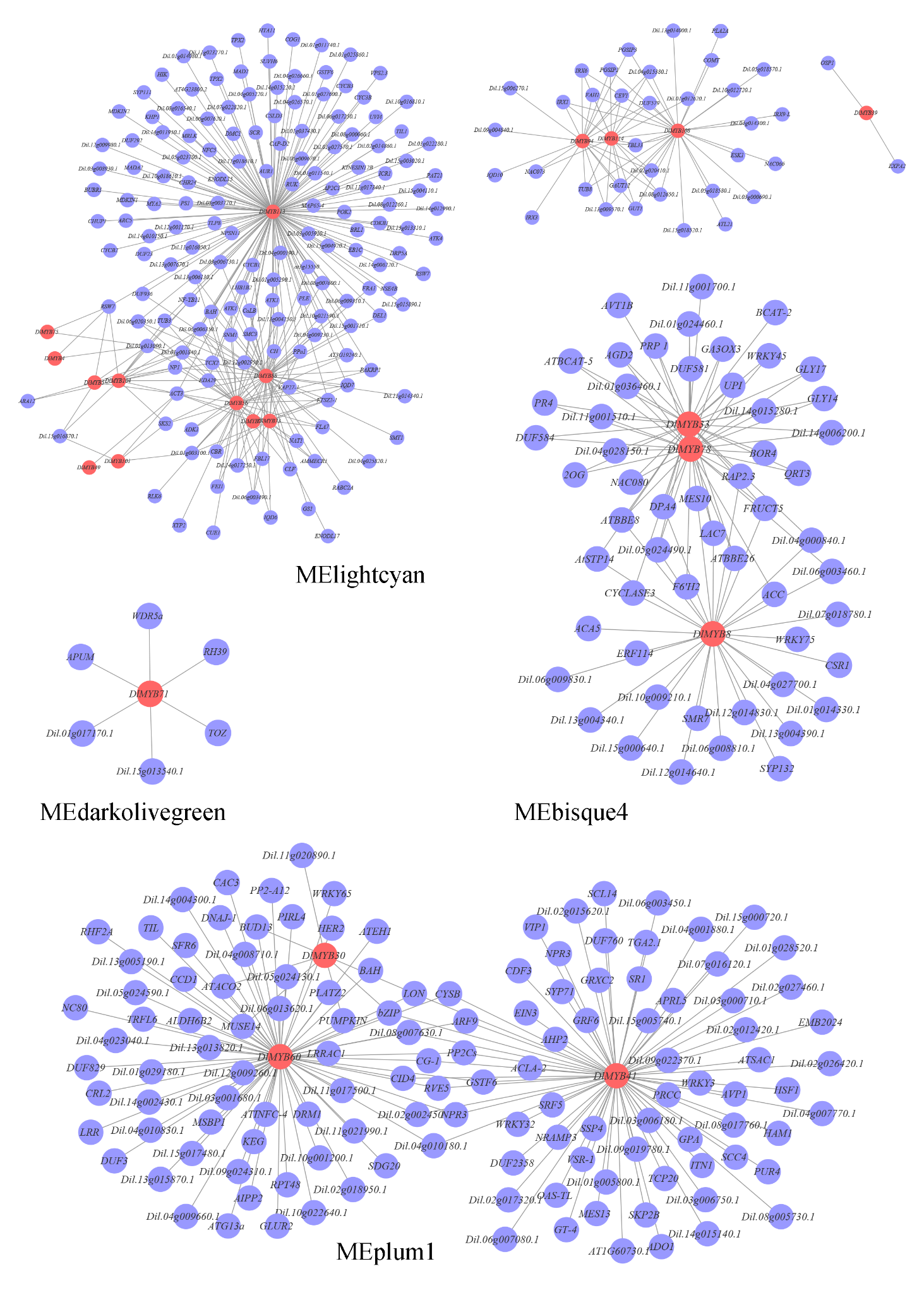
**

**Figure S8 Co-expression networks of differentially expressed *R2R3-MYB* genes during off-season flower induction by KClO_3_.** Each node represents a gene and the lines between nodes represent co-expression correlations.


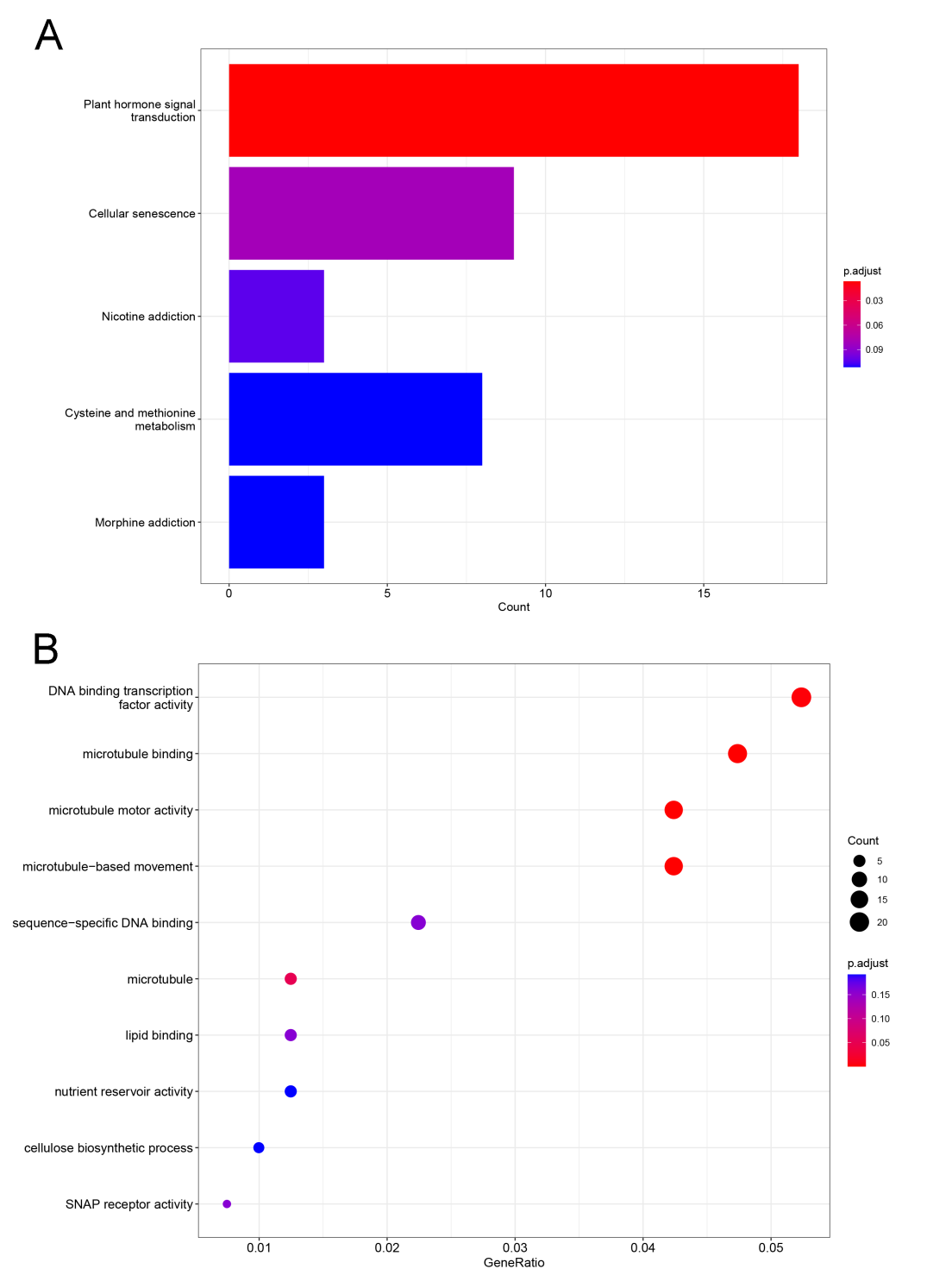


**Figure S9 KEGG (Kyoto Encyclopaedia of Genes and Genomes) pathway enrichment analysis and Gene ontology (GO) classification of differentially expressed genes (DEGs) that had interactions with *DlMYB* genes.**
